# Seed-Borne *Erwinia persicina* Affects the Growth and Physiology of Alfalfa (*Medicago sativa* L.)

**DOI:** 10.3389/fmicb.2022.891188

**Published:** 2022-05-26

**Authors:** Bo Yao, Rong Huang, Zhenfen Zhang, Shangli Shi

**Affiliations:** Key Laboratory of Grassland Ecosystem, Ministry of Education, Sino-U.S. Centers for Grazing Land Ecosystem Sustainability, Ministry of Science and Technology, College of Grassland Science, Gansu Agricultural University, Lanzhou, China

**Keywords:** *Erwinia persicina*, alfalfa, plant-microbe interaction, growth, physiology

## Abstract

Seed-borne *Erwinia persicina* can be transmitted globally via alfalfa (*Medicago sativa* L.) seed trade, but there is limited information about the impact of this plant-pathogenic bacterium on alfalfa plants. In this study, strain Cp2, isolated from alfalfa seeds, was confirmed by whole-genome sequencing to belong to *E. persicina*. Subsequently, the effects of Cp2 on alfalfa growth and physiology were evaluated by constructing a rhizosphere infection model. Strain Cp2 had a strong inhibitory effect on the elongation and growth of alfalfa roots, which was very unfavorable to these perennial plants. Furthermore, an increased number of leaf spots and yellowing symptoms were observed in plants of the Cp2 group from day 10 to day 21 and the strain Cp2 was re-isolated from these leaves. Correlation between growth and photosynthetic parameters was analyzed and the significant decreases in fresh weight and root and plant lengths in the Cp2 group were related to the marked reduction of chlorophyll *b*, carotenoid, transpiration rate, and stomatal conductance of leaves (*r* > 0.75). In addition, nine physiological indicators of root, stem, and leaf were measured in the plants 21 days after treatment with Cp2. The physiological response of root and leaf to Cp2 treatment was stronger than that of stem. The physiological indicators with the greatest response to Cp2 infection were further explored through principal component analysis, and superoxide dismutase, peroxidase, ascorbate peroxidase, and soluble protein showed the greatest changes in roots, stems, and leaves (*P* < 0.001). Among tissues, the commonality was the change of soluble protein. Therefore, soluble protein is speculated to be a physiological marker during alfalfa–*E. persicina* interactions. These findings indicate that once *E. persicina* spreads from alfalfa seeds to the rhizosphere, it can invade alfalfa roots and cause disease. This study demonstrates that this plant pathogenic bacterium may be a potential threat to new environment when they spread via seed trade and these “dangerous hitchhikers” warrant further attention, especially in the study of bacterial diseases in pasture-based production systems.

## Introduction

Alfalfa (*Medicago sativa* L.), a perennial plant, is the most widely planted legume forage in sustainable animal husbandry due to its high nutritional and ecological value; it is cultivated in more than 80 countries, encompassing every continent of the globe in an area exceeding 35 million ha (Radović et al., 2009; Moot et al., 2012). One of the factors limiting alfalfa production is pathogenic bacteria (Nan, 2001). *Erwinia persicina* (*E. persicina*), a pathogenic bacterium, can infect alfalfa and cause sprout wilting (germination test) and leaf spot (spray inoculation) (Zhang and Nan, 2014a). *E. persicina* is a seed-borne bacterium and can be isolated from *Anemone rivularis*, *Elymus nutans*, and turfgrass seeds (Ban et al., 2021; Liang et al., 2021). In our previous study, *E. persicina* is a predominant species isolated from alfalfa seeds (Zhang, 2013). Alfalfa seeds provide a “home” for *E. persicina*, and the increasing frequency of trading of alfalfa seeds greatly facilitates the spread of pathogens with the seeds to new environments (Kimball, 2016; Shi et al., 2017). However, there are limited studies about the effect of seed-borne *E. persicina* on alfalfa plant.

*Erwinia persicina*, gram-negative, rod-shaped bacterium, is a member of the genus *Erwinia* in the family *Erwiniaceae*, and was characterized by its ability to produce a water-soluble pink pigment (Adeolu et al., 2016). There is only a short history of research on *E. persicina*. The bacterium was first isolated from tomatoes, bananas, and cucumbers by Komagata et al. in 1963, but was not officially named until 1990 (Hao et al., 1990). *E. persicina* is a phytopathogenic bacterium causing a variety of plant diseases in various hosts. The first report on the pathogenicity of *E. persicina* was the disease of beans (*Phaseolus vulgaris* L.) described by Schuster et al. (1981) in the United States. Symptoms of this disease were yellow spots initially that later became irregular necrotic lesions on the leaves, while another striking symptom was the pink discoloration of white bean seed (Schuster et al., 1981; Brenner et al., 1994). In Spain, chlorotic and necrotic lesions on leaves and tendrils infected by *E. persicina* were observed in bean (*Phaseolus vulgaris* L.) and pea (*Pisum sativum* L.), which caused losses as much as 50% and affected approximately 12 ha (González et al., 2005, 2007). In addition to infecting legumes, *E. persicina* also causes diseases of *Cucurbitaceae* (cucumber, melon, and zucchini), *Solanaceae* (tomato), *Liliaceae* (garlic), *Apiaceae* (parsley and celery), *Asteraceae* (lettuce), and *Poaceae* (barley) plants (Diánez et al., 2009; Gálvez et al., 2015; Nechwatal and Theil, 2019; Orel, 2020; Kawaguchi et al., 2021; Li et al., 2021; Wang et al., 2021). The survival ability of strains of *E. persicina* have made this bacterium highly adaptable to different environments. *E. persicina* has been reported to inhabit the intestinal tracts of river trout, gilthead sea bream, diamondback moth, and redbanded stink bug (Skrodenyte-Arbaciauskiene et al., 2006; Floris et al., 2013; Li et al., 2016; Husseneder et al., 2017). Moreover, the species was also found in extremely desiccated and low-temperature environments (Kiessling et al., 2005; Baruzzi et al., 2012). Thus, *E. persicina* has a wide range of hosts and strong adaptability capacity.

Infection of plants by bacterial pathogens cause deleterious shifts in the physiological and biochemical processes required for normal cellular reproduction, function, and differentiation (Daly, 1976). At the physiological level, the interaction between plants and pathogenic bacteria is predominantly manifested in osmotic adjustment, photosynthesis, oxidation and antioxidation, and synthesis of defense enzymes (Liang et al., 2006). Osmotic adjustment plays a part in the regulation of the nutrient supply available to invading bacteria, so a change in osmoregulation is the initial response of plants when pathogenic bacteria colonize interior portions of plant tissues, and soluble substances play a role in osmotic adjustment to protect plants from stress (Wheeler and Hanchey, 1968; Zhao et al., 2021). Photosynthesis dominates plant energy metabolism, which helps with the synthesis of defense molecules against pathogenic bacteria, and pathogens have evolved the ability to interfere and regulate photosynthesis to facilitate infection (Lu and Yao, 2018). Reactive oxygen species (ROS) are instrumental in the interaction between plants and pathogens.

Reactive oxygen species are regarded as cellular signaling molecules that activate plant immunity under normal conditions, but excessive ROS damage plant cells through various processes including lipid peroxidation, protein oxidation, oxidative damage to DNA, and monosaccharide oxidation. Plants have evolved effective mechanisms to respond to oxidative damage by using antioxidant enzymes, including superoxide dismutase (SOD), catalase (CAT), peroxidase (POD), and ascorbate peroxidase (APX), to neutralize the negative effects of ROS (Dumanović et al., 2021). When bacterial pathogens continue to infect hosts, plants can activate defense enzymes that are related to the synthesis of secondary metabolites, including polyphenol oxidase (PPO) and phenylalanine ammonia lyase (PAL), which are involved in the synthesis of phenols, quinones, flavonoids, lignin, and other secondary defense substances (Ngadze et al., 2012). Studying the physiological response of hosts to pathogens has enhanced understanding of the pathogenic mechanisms involved in these interactions. However, there is limited research on bacterial disease(s) of alfalfa, with only the pathogenic phenomena reported (Zhang and Nan, 2014b), and there are currently gaps in the research and knowledge on the physiological response of alfalfa to pathogenic bacteria.

This study aimed to explore the effects of *E. persicina* isolated from alfalfa seeds on alfalfa plants. Strain Cp2 was identified as *E. persicina* based on phylogenetic analysis of 16S rRNA sequences in our previous study (Zhang, 2013) and this was confirmed by whole genome sequencing in the current study. A model of *E. persicina* infecting alfalfa plants from the roots was then constructed and used to evaluate the significant effects of *E. persicina* on roots and leaves based on growth and physiological indicators. To our knowledge, this is the first study to explore physiological changes during alfalfa bacterial diseases and lays a foundation for the study of the pathogenic mechanism(s) of *E. persicina*.

## Materials and Methods

### Cultivation of Bacterial Strains and DNA Extraction

The bacterial strain selected for this research was Cp2, which was isolated from the internal tissue of alfalfa seeds and stored at −80°C (Zhang, 2013). Strain Cp2 was resuscitated on nutrient agar (NA) solid medium at 30°C. After culture for 24 h, single colonies were inoculated into nutrient broth (NB) and incubated for 48 h at 30°C on a rotary shaker at 160 rpm. Bacterial cells were then harvested by centrifugation at 5,000 rpm for 15 min and genomic DNA was extracted from the cells using a Bacterial Genomic DNA Extraction Kit (TIANGEN, Beijing, China).

### Whole Genome Sequencing, Assembly, and Annotation

Concentration and purity of the genomic DNA were quantified with TBS-380 Mini-Fluorometer (Turner BioSystems) and NanoDrop 2500, respectively. The genome of strain Cp2 was sequenced by Shanghai Majorbio Bio-Pharm Technology Co., Ltd., Shanghai, China using Illumina HiSeq and PacBio methods. After sequencing, clean reads were obtained from the raw reads by removing adaptor sequences, reads containing low-quality reads and poly-N. The resulting clean reads were used for scaffolding by SOAPdenovo software. High-quality PacBio long reads were assembled by using Canu (Koren et al., 2017). After assembly, circular chromosomes and plasmids with gapless were finally generated and drawn using the CGView program (Stothard and Wishart, 2005). Prediction of genes for protein coding sequences (CDSs), transfer RNA (tRNA), and ribosomal RNA (rRNA) was performed with Glimmer, GeneMarks, tRNAscan-SE, and Barrnap software, respectively (Besemer et al., 2001; Seemann, 2013; Lowe and Chan, 2016). In addition, each predicted CDS was annotated through the Clusters of Orthologous Genes (COG) database by BLAST (Tatusov et al., 2003). Genes associated with virulence were identified in the Virulence Factors Database (VFDB) and Pathogen-Host Interaction Database (PHI-base) (Chen et al., 2005; Winnenburg et al., 2006).

### Average Nucleotide Identity Analysis

Strain Cp2 was identified as belonging to the genus *Erwinia* based on results from our previous work (Zhang, 2013). To further determine taxonomic positioning, ANI values between whole genomes of strain Cp2 and other sequenced *Erwinia* strains were calculated using JSpeciesWS^1^. Twenty-three whole genome sequences of strains of *Erwinia* were downloaded from the NCBI Genome database^2^. An ANI value of 95% was used as the boundary for species delineation. Finally, a heatmap based on ANI values was generated by using TBtools software.

### Pan-Genome Analysis

The pan-genome of *E. persicina* strain Cp2 and nine other *E. persicina* strains was performed using PGAP pipeline (Zhao et al., 2012). The Gene Family (GF) method was used to search for the orthologs among these 10 strains. Pan and core genome curve were visualized by using GraphPad Prism version 9. A Venn diagram to represent the core and unique genes was drawn by using the online tool^3^.

### Inoculation Assay

*Erwinia persicina* Cp2 was cultured in NB for 24 h at 30°C, then the bacterial cells were pelleted by centrifugation at 8,000 rpm for 5 min and were resuspended in sterile water. Next, a Cp2 suspension at a concentration of 10^9^ CFU/mL was prepared. CFU/mL values were calculated based on the standard curve of optical density obtained at 600 nm (OD_600_) for the Cp2 suspension.

Experiments featured healthy and full seeds of alfalfa (*Medicago sativa* L.; provided by the Sino-U.S. Centers for Grazing Land Ecosystem Sustainability) and were divided into *E. persicina*-treated (Cp2 group) and control (CK group) groups. Glass transparent growth bottles (Chinese Utility Patent; patent number: ZL-2020-2-1672986.4) were used for plant-growth experiments. The growth bottle has two characteristics: first, after 200 mL solution is loaded into the bottle, the liquid surface just contacts the undersurface of the germination bed, which ensures that the seeds absorb water normally; second, the cover of the growth bottle has a filter membrane structure, which can exchange gas but filter out bacteria.

Before inoculation, alfalfa seeds were surfaced-sterilized for 2 min in 75% (v/v) ethanol and then 10 min in 5% (v/v) sodium hypochlorite, followed by washing five times with sterile water. Solutions (200 mL/bottle) of the treatment group (Cp2 bacterial suspension) and the control group (sterile water) were then separately loaded into the growth bottles. The formal inoculation experiment was performed in a biosafety cabinet. Alfalfa seeds were placed equidistant (seed spacing = 1 cm) on the germination bed of the growth bottle (25 seeds/bottle) with tweezers. All articles used in the inoculation experiment were sterilized prior to use, and the entire process was performed under sterile conditions. After inoculation, growth bottles were placed in a growth chamber at 23°C day and 20°C night temperature, 16-h day and 8-h night photoperiod. Germination and growth were observed and recorded daily.

### Measurement of Growth-Related Indicators

Seed germination indicators such as germination rate (GR %), germination potential (GP %), and germination index (GI) were recorded for seven consecutive days. The calculation formulae were as follows: GR = total germinated seeds/25 × 100%; GP = the germination number on the 3rd day/25 × 100%; GI = ΣGt/Dt, where Gt = germination number at different times (7 days) and Dt = number of days of germination. After 21 days, basic growth indicators including plant height and fresh and dry weights were measured, the Expression 12000XL root system scanning analyzer (Seiko Epson Corporation, Suwa, Nagano, Japan) was used to scan roots, and the morphological index (e.g., root diameter) was analyzed by WinRHIZO Basic 2013. The root water potential (RWP) and ambient water potential (AWP) of CK and Cp2 group were further performed using a Dewpoint PotentiaMeter (WP4C, Meter Group, Pullman, WA, United States).

Leaf chlorophyll content was determined using ethanol solvents according to literature (Yu et al., 2019) with slight modifications. Briefly, leaf samples (0.1 g) of Cp2 and CK groups were soaked in 95% alcohol in a 5-mL tube, then each tube was wrapped in tinfoil and placed at room temperature for 24 h. Absorbance readings at 470, 665, and 649 nm for the collected supernatants were used to estimate contents of carotenoids, chlorophyll *a*, and chlorophyll *b*, respectively. The contents of chlorophyll *a*, chlorophyll *b*, and carotenoids (all in units of mg/g) were calculated according to the formulae: chlorophyll *a* content (Chla) = (13.95 × A665–6.88 × A649) × 5 × 10^–3^/FW; chlorophyll *b* content (Chlb) = (24.96 × A649–7.32 × A665) × 5 × 10^–3^/FW; total chlorophyll content (ChlT) = Chla + Chlb; carotenoids content (Car) = (1000 × A470–2.05 × Chla–114.8 × Chlb) × 5 × 10^–3^/245 × FW. A470, A665, and A649 represent the absorbance value at 470, 665, and 649 nm, respectively; FW is fresh weight of leaves.

### Determination of Photosynthetic Parameters of Leaves

Photosynthetic parameters of the leaves of plants in the Cp2 and CK groups were measured by using a portable photosynthesis system (GFS-3000, Heinz Walz GmbH, Effeltrich, Germany). Net photosynthesis rate (Pn), stomatal conductance (Gs), intercellular CO_2_ (Ci), and transpiration rate (Tr) were determined at 380 μmol/m^2^/s CO_2_ and 1,200 μmol/m^2^/s photosynthesis photon flux density (PPFD). Measurements were performed in the mornings between 9:00 a.m. and 11:00 a.m. on day 21. The photosynthetic parameters (Pn, Ci, Gs, and Tr) were calculated according to Von Caemmerer and Farquhar (1981).

### Antioxidant Enzyme Assays

Roots, stems, and leaves of plants in the Cp2 and CK groups were harvested at day 21 and used to determine activities of antioxidant enzymes, including SOD, CAT, POD, and APX. The four antioxidant indicators were determined according to the protocol of SOD (M0102A), CAT (M0103A), POD (M0105A), and APX (M0403A) assay kits (Suzhou Michy Biomedical Technology Co., Ltd, Suzhou, China), respectively. Briefly, samples (0.1 g) were homogenized in extraction buffer, centrifuged at 12,000 rpm for 10 min at 4°C, and the resulting supernatants were collected for determination of enzyme activities using the corresponding assay kits.

### Measurement of Malondialdehyde Content

Malondialdehyde content, an indirect measurement of lipid peroxidation, was determined using an MDA (M0106A) assay kit (Suzhou Michy Biomedical Technology Co., Ltd, Suzhou, China). The absorbance value of the supernatant was measured at 532 nm by using a microplate reader (Spectra Max iD3, Molecular Devices, United States) and the content of MDA was expressed as nmol/g FW.

### Estimation of Soluble Protein and Soluble Sugar Contents

Soluble protein and soluble sugar contents of roots, stems, and leaves were determined using BCA protein (M1806A) and soluble sugar (M1503A) assay kits (Suzhou Michy Biomedical Technology Co., Ltd, Suzhou, China), respectively. Measurement of SP content utilized the bicinchoninic acid (BCA) method. The sample (0.1 g) was mixed with 1 mL extraction buffer and then centrifuged at 12,000 rpm for 10 min at 4°C. The resulting supernatant was analyzed for SP using the BCA working solution in the kit and measuring the absorbance at 562 nm. SP content in each sample was determined by comparing the absorbance reading to those of bovine serum albumin (BSA) standards. Measurement of SS content was based on the anthrone method (Buysse and Merckx, 1993), with the absorbance determined at 620 nm using an ultraviolet spectrophotometer.

### Quantification of Phenylalanine Ammonia Lyase and Polyphenol Oxidase

Assays for PAL and PPO activities were performed in alfalfa roots, stems, and leaves 21 days post-inoculation. PAL and PPO activities were determined according to the methods of Kar and Mishra (1976) and Aydaş et al. (2013), respectively. Briefly, each sample was homogenized with extraction buffer at the ratio of 0.1 g/1 mL and was centrifuged at 12,000 rpm for 10 min at 4°C. The resulting supernatant was used for the determination of PAL and PPO by PAL (M0110A) and PPO (M0109A) assay kits (Suzhou Michy Biomedical Technology Co., Ltd, Suzhou, China).

### Statistical Analysis

Statistical analyses were performed using R and SPSS software. Correlation analysis was performed using Spearman’s correlation. Principal component analyses (PCA) were performed using the R “*prcomp*” function. The heatmap was generated using TBtools software.

## Results

### General Genome Overview and Average Nucleotide Identity Analysis of Strain Cp2

**FIGURE 1:**
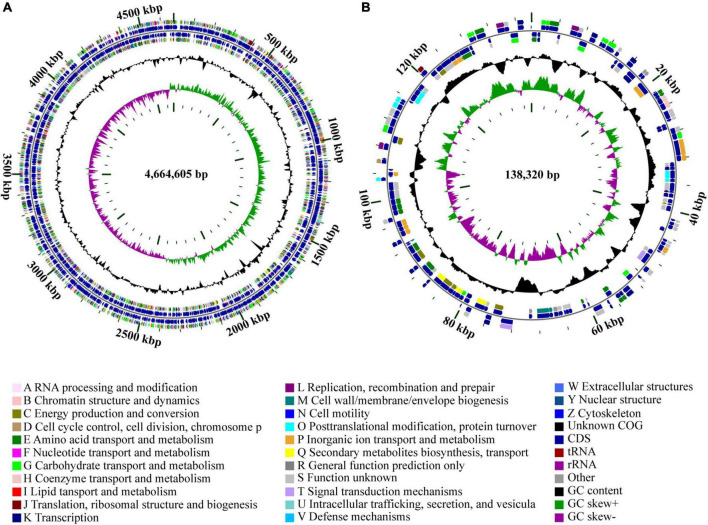
Circular genome maps of **(A)** chromosome and **(B)** plasmid of strain Cp2. The circles from the inside to outside represent: (1) GC skew; (2) GC content; (3) CDS on the reverse strand was annotated by COG database; (4) CDS, rRNA, and tRNA on the reverse; (5) CDS, rRNA, and tRNA on the forward; (6) CDS on the forward; (7) scale of genome size.

**FIGURE 2:**
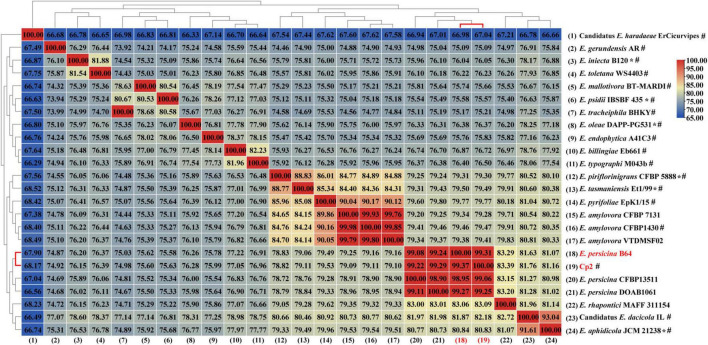
A heat map of average nucleotide identify (ANI) based on the whole genome sequence of strain Cp2 and 23 strains within genus *Erwinia.* *Represents type strain, ^#^represents reference genome in the NCBI genome database.

The genome of strain Cp2 comprised a single circular chromosome of 4,664,605 bp with 55.53% GC content and one plasmid of 138,320 bp with 55.52% GC content (Figure 1). The circular chromosome contained 4,410 CDS, 81 tRNA, and 22 rRNA genes, while the plasmid had 152 CDS. The protein-coding genes were assigned to 25 COG functional categories (Figure 1). Our previous work identified strain Cp2 as *Erwinia* spp. by 16S rRNA gene sequencing (Zhang, 2013). To clarify the phylogenetic relationship between Cp2 and related species of the genus *Erwinia*, an ANI analysis based on BLAST was conducted. ANI values between strain Cp2 and 23 species of the genus *Erwinia* ranged from 67.04 to 99.37% (Figure 2). Strain Cp2 was closely related to *Erwinia persicina* with ANI values ranging from 98.95 to 99.37%, which exceeded the ANI species threshold of 95%. Thus, based on 16S rRNA gene sequencing and ANI analyses, strain Cp2 was identified as *Erwinia persicina* and was designated as *Erwinia persicina* Cp2.

### Potential Virulence Genes in Strain Cp2 Genome

**FIGURE 3:**
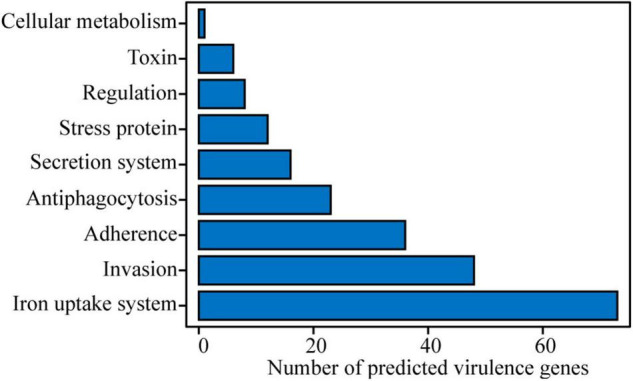
Potential virulence factors in *E. persicina* strain Cp2 genome predicted by VFDB database.

Based on a BLAST search in VFDB, potential virulence genes were identified. A total of 509 putative virulence genes were aligned in strain Cp2 genome with 493 in the chromosome and 16 in the plasmid (Supplementary Table 1). Among them, 223 genes had annotation information in VFDB database, these genes were classified into nine categories, including cellular metabolism (1 gene), toxin (6 genes), regulation (8 genes), stress protein (12 genes), secretion system (16 genes), antiphagocytosis (23 genes), adherence (36 genes), invasion (48 genes), and iron uptake system (73 genes) (Figure 3). We also obtained a total of 783 annotated genes by using the PHI-base (Supplementary Table 2). Among them, *iaaH* genes involved in indole-3-acetic acid (IAA) biosynthesis were found in the *E. persicina* Cp2 genome.

### Pan-Genome Analysis of *E. persicina* Strains

**FIGURE 4:**
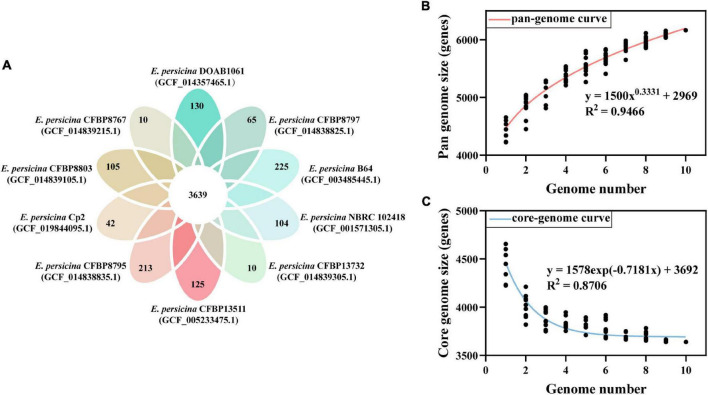
Pan-genome and core-genome of *E. persicina.* **(A)** Petal diagram of homologous genes of *E. persicina.* The numbers of core genes in 10 *E. persicina* strains was represented in the center and strain-specific genes in the petals. Latest RefSeq assembly accession numbers for each strain is presented in parentheses. **(B)** Pan-genome plot of *E. persicina.* The curve, fitted by the power-law regression model, represents the relationship between the number of genome and pan-genome size. **(C)** Core-genome plot of *E. persicina.* The curve, fitted by exponential curve fit model, indicates the number of core-genome as a function of the number of sequentially added genomes.

The PGAP analysis revealed that the 10 compared *E. persicina* strains encompasses a total number of 6,165 genes. Of these, 3,639 (59.03% of total pan-genome) were core genes across all 10 strains genomes. After comparing strain-specific genes, we found that the number of *E. persicina* strain-specific genes varied from 10 to 225 genes, with strain Cp2 having 42 specific genes (Figure 4A). The pan-genome curve was performed using a power law regression, and the function, *y* = 1500 *x*^0.3331^ + 2969, revealed that the pan-genome of *E. persicina* had a parameter (γ) of 0.3331, and α (=1−γ) < 1 represented that the pan-genome would continuously increase as more *E. persicina* genomes are sampled (Li et al., 2019; Figure 4B). Furthermore, the core-genome curve, *y* = 1578 exp (−0.7181 *x*) + 3692, revealed that the addition of an extra *E. persicina* genome would not significantly alter the core genome size due to numbers of core genes were relatively stable (Figure 4C).

### Effect of *E. persicina* Cp2 on the Growth of Alfalfa

**FIGURE 5:**
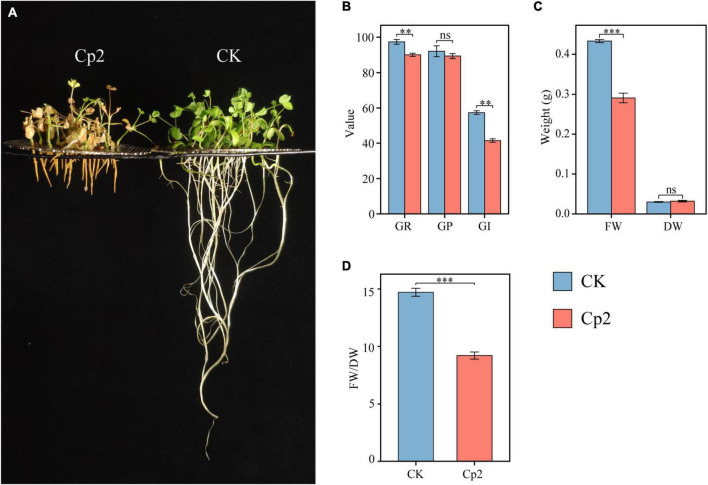
Alfalfa phenotypes and growth indicators at 21 days after inoculation with *E. persicina* Cp2. **(A)** Phenotypic comparison between CK and Cp2 groups. **(B)** Comparison of germination between CK and Cp2 (Kruskal–Wallis test, Dunn’s test, *n* = 6). GR (%), germination rate; GP (%), germination potential; GI, germination index. **(C)** Fresh and dry weight compared to the CK (Independent samples *t*-test). FW, fresh weight; DW, dry weight. **(D)** FW/DW: fresh weight/dry weight matter (Mann–Whitney *U* test). ns: *P* ≥ 0.05, ^**^P < 0.01, ^***^P < 0.001; *n* = 6; mean ± SE.

Compared with the control (CK group), the growth of alfalfa was significantly inhibited after inoculation with *E. persicina* Cp2 (Figure 5A). During seed growth, the germination rate and germination index of the Cp2 group were significantly lower compared with those of the CK group (*P* < 0.01) (Figure 5B). After growth for 21 days, the fresh weight of alfalfa in the Cp2 group was much less than that in the CK group (*P* < 0.01), while no significant differences in dry weight (Figure 5C). From the fresh-dry weight ratio, it was concluded that the water content of alfalfa plants was reduced in the Cp2 treatment group compared with the CK group (Figure 5D).

### Root Growth of Alfalfa Was Significantly Inhibited After Inoculation With *E. persicina* Cp2

**FIGURE 6:**
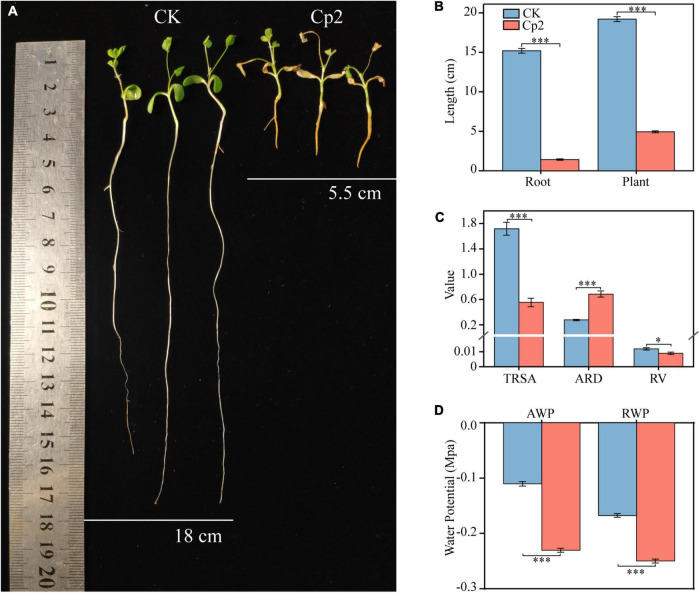
Inhibition of alfalfa root elongation and growth by *Erwinia persicina* strain Cp2. **(A)** The picture was taken at 21 days postinoculation. **(B)** Changes of root and plant lengths between CK and Cp2 group (Mann–Whitney *U* test, *n* = 9). **(C)** Differences in the root growth parameters between CK and Cp2 group (Welch *t*-test, *n* = 6). TRSA, total root surface area (cm^2^); ARD, average root diameter (mm); RV, root volume (cm^3^). **(D)** Water potential of CK and Cp2 group on day 21 (Independent samples *t*-test, *n* = 12). AWP, ambient water potential; RWP, root water potential. *P < 0.05, ^***^P < 0.001; mean ± SE.

*Erwinia persicina* Cp2 demonstrated a strong inhibitory effect on the growth of alfalfa roots. Plant length in the Cp2 group was significantly less compared with that of the CK group (Figure 6A), which was mainly due to the strong inhibition of root elongated growth of the Cp2 group (*P* < 0.001) (Figure 6B). Growth parameters that characterize the absorption of water and nutrients by plants were subsequently analyzed (Figure 6C). Compared with plants in the CK group, the total root surface area (*P* < 0.001) and root volume (*P* < 0.1) of alfalfa in the Cp2 group were decreased, while the average root diameter was significantly increased (*P* < 0.001). In summary, the roots became shorter and thicker during the interaction between *E. persicina* Cp2 and alfalfa, which negatively impacts water and nutrient absorption in the roots of alfalfa. To determine the effect of Cp2 suspension on alfalfa’s water uptake, we further analyzed the water potential of root and ambient in CK and Cp2 group. Compared with the CK group, the AWP (Cp2 suspension) was decreased at day 21. However, the RWP was still below the AWP in Cp2 group (Figure 6D). This suggests that *E. persicina* Cp2 has little effect on root water uptake.

### Observation of Leaf Symptoms and Determination of Photosynthetic Physiology After Inoculation With *E. persicina* Cp2

**FIGURE 7:**
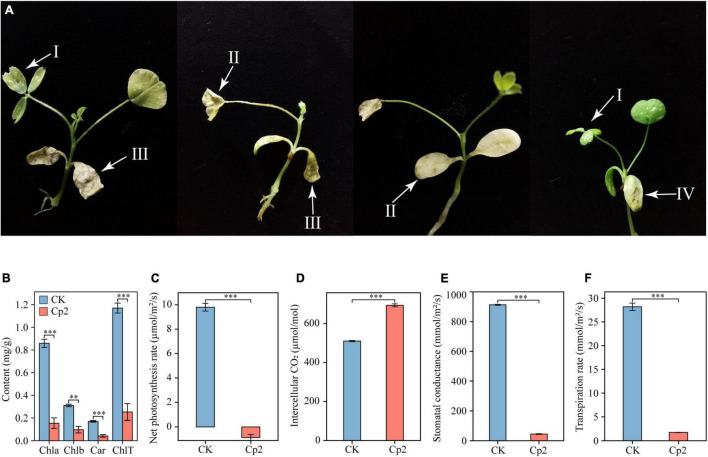
Symptoms on alfalfa leaves **(A)** caused by *E. persicina* Cp2. These pictures were taken 21 days. I, leaf spots; II, chlorosis; III, leaf wilting; IV, necrosis. **(B)** Differences in the chlorophyll content of alfalfa leaves in the CK and Cp2 groups (One-way ANOVA test, Tukey’s HSD test, *n* = 6). Chla, chlorophyll *a*; Chlb, chlorophyll *b*; Car, carotenoid; ChlT, total chlorophyll. **(C)** Net photosynthesis rate. **(D)** Intercellular CO_2_ concentration. **(E)** Stomatal conductance. **(F)** Transpiration rate. Independent samples *t*-test, ns: *P* ≥ 0.05, *P < 0.01, ^***^P < 0.001; *n* = 6; mean ± SE.

Alfalfa leaves exhibited certain symptoms following inoculation with *E. persicina* Cp2. Leaf spots, leaf wilting, necrosis, and chlorosis were visible on the leaves at day 21 after inoculation (Figure 7A). To validate Koch’s postulates, *E. persicina* Cp2 were re-isolated from the leaves of Cp2 group by selective media (NA plates containing 100 μg/mL ampicillin) (O’Hara et al., 1998; Zhang and Nan, 2014a). We speculated that strain Cp2 invades alfalfa from the root, then moves vertically up the stem, and finally colonizes the leaves and causes disease. To test this, strains Cp2 were further re-isolated from the roots and stems by using the same approach.

Measurement of the photosynthetic physiology of leaves revealed that, compared with the control group, the chlorophyll *a*, chlorophyll *b*, and carotenoid contents of the Cp2 group were significantly reduced, and the total chlorophyll content was reduced by 78.39% (Figure 7B). The net photosynthetic rate (Pn), stomatal conductance (Gs), and transpiration rate (Tr) of the leaves in the Cp2 group were significantly reduced compared with those of the CK group, while the intercellular carbon dioxide concentration (Ci) was significantly increased (Figures 7C–F). Among these parameters, the Pn was negative in the Cp2 group, indicating that the photosynthetic activity of the mesophyll cells had decreased.

### Correlation Analysis Between Growth and Photosynthetic Physiology Indicators

**FIGURE 8:**
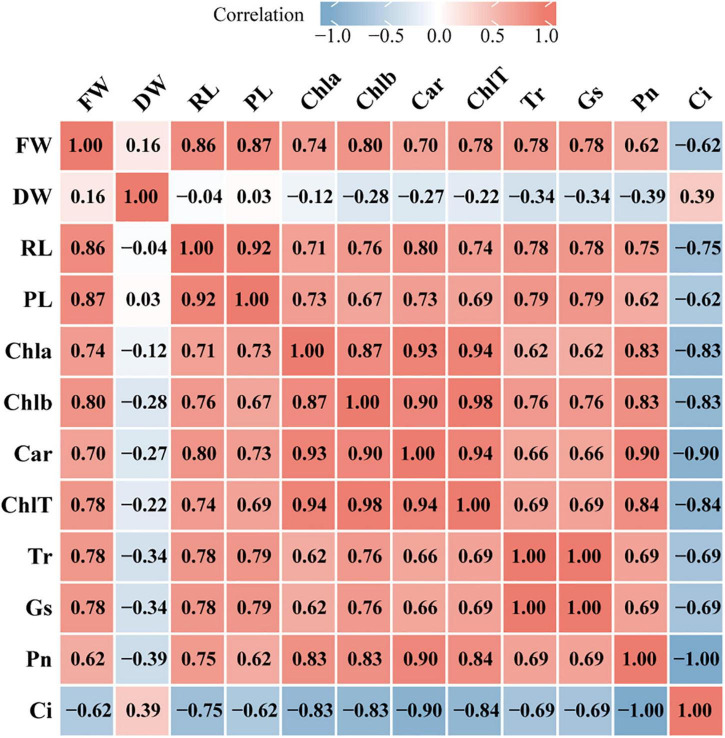
Heatmap of correlation between growth indicators and photosynthetic physiology indicators. The image show Spearman correlation and the values in the grid represent correlation coefficients (*r* values). The red color indicates a positive (0 < *r* < 1.0) correlation and the blue color indicates a negative (–1.0 < *r* < 0) correlation. Strength of correlation: absolute values of *r* > 0.7 indicate strong correlation, 0.4 < absolute values of *r* ≤ 0.7 indicate moderate correlation, absolute values of *r* ≤ 0.4 indicate weak correlation. FW, fresh weight; DW, dry weight; RL, root length; PL, plant length; Chla, chlorophyll *a*; Chlb, chlorophyll *b*; Car, carotenoid; ChlT, total chlorophyll; Tr, transpiration rate; GS, stomatal conductance; Pn, net photosynthesis rate; Ci, intercellular CO_2_ concentration.

To explore the relationship between plant growth and photosynthesis, the correlation among growth indicators (FW, fresh weight; DW, dry weight; RL, root length; PL, plant length) and photosynthetic physiology indicators (Chla, chlorophyll *a*; Chlb, chlorophyll *b*; Car, carotenoid; ChlT, total chlorophyll; Tr, transpiration rate; Gs, stomatal conductance; Pn, net photosynthesis rate; Ci, intercellular CO_2_ concentration) were analyzed using Spearman’s correlation test. FW was strongly positively correlated with chlorophyll contents, Tr, and Gs (*r* > 0.75) but moderately negatively correlated with Ci (*r* = −0.62), while DW was weakly positively correlated with Ci (*r* = 0.39) (Figure 8). In addition, most of the photosynthetic physiology indicators were positively correlated with PL and RL except Ci. Furthermore, chlorophyll contents were significantly associated with Pn and Ci, for instance, ChlT had a strong positive relationship with Pn (*r* = 0.84) but a negative relationship with Ci (*r* = −0.84). The correlation analysis also demonstrated a strong negative correlation between Pn and Ci (*r* = −1) and a significant positive correlation between Tr and Gs (*r* = 1).

### Comparative Analysis of Physio-Biochemical Indicators Measured in the CK and Cp2 Groups

**FIGURE 9:**
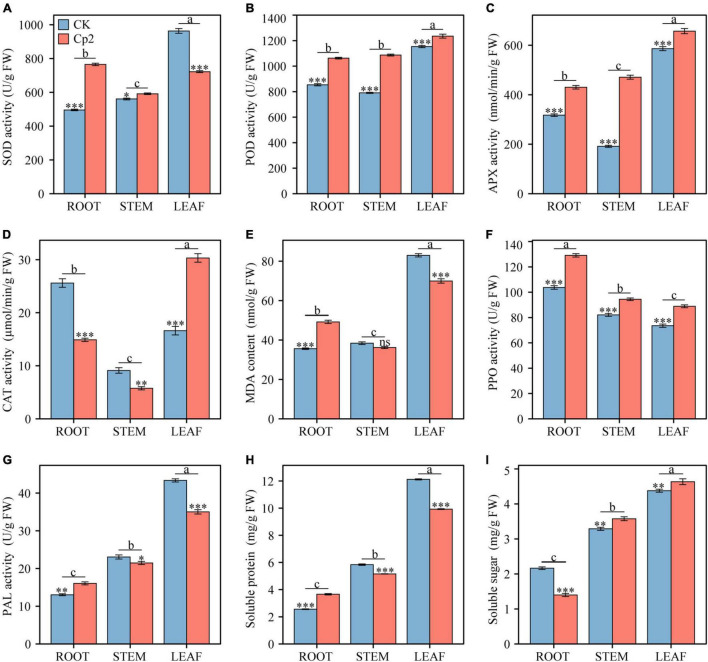
Comparison of nine physiological indicators between different treatments and different plant tissues after 21 days of inoculation with *E. persicina* Cp2. **(A)** superoxide dismutase (SOD) activity. **(B)** peroxidease (POD) activity. **(C)** ascorbate peroxidase (APX) activity. **(D)** catalase (CAT) activity. **(E)** malondialdehyde (MDA) content. **(F)** polyphenol oxidase (PPO) activity. **(G)** phenylalanine ammonia lyase (PAL) activity. **(H)** soluble protein. **(I)** soluble sugar. FW, fresh weight. Two-way ANOVA test, Bonferroni post test. CK versus Cp2: ns: *P* ≥ 0.05, *P < 0.05, **P < 0.01, ***P < 0.001; root versus stem versus leaf: a–c, *P* < 0.01. n = 3; mean ± SE.

After 21 days of growth, nine physio-biochemical indicators of alfalfa were measured in different plant tissues (root, stem, and leaf) under different treatment groups (CK and Cp2 groups), and the results were analyzed by two-way ANOVA with Bonferroni correction (Supplementary Table 3). First, the antioxidant enzyme system activity changes, including SOD, APX, POD, and CAT were examined (Figures 9A–D). SOD activity increased in the roots and stems of alfalfa in the Cp2 group compared with that of the CK group but decreased in the leaves. The variation tendency of the CAT enzyme was opposite to SOD, declining in the roots and stems but increasing in the leaves. POD and APX activities showed the same significantly increasing tendency in roots, stems, and leaves of alfalfa in the Cp2 group compared with that of the CK group. Thus, significant differences were observed in four antioxidant enzyme indicators between roots, stems, and leaves of the Cp2 group compared with the CK group, and the activities of the four antioxidant enzymes were higher in leaves compared with roots and stems. MDA is one of the most important end products of membrane lipid peroxidation induced by a diversity of oxidative injuries, therefore the MDA content in roots, stems, and leaves was also measured. MDA content in alfalfa of the Cp2 group was significantly increased in roots, decreased in leaves, and exhibited no significant difference in stems compared with the CK group, and its content was markedly higher in the leaves compared with that in the roots and stems (Figure 9E).

The activities of defense enzymes, including PAL and PPO, in the alfalfa under pathogenic stress was subsequently evaluated. PPO activity increased significantly in roots, stems, and leaves of the Cp2 treatment group compared with that of the CK group, while PAL enzyme activity increased considerably in the roots but decreased in leaves and stems (Figures 9F,G). These two enzymes both exhibited significant differences in activity in different plant tissues following inoculation with strain Cp2, but the change trend of their activities in roots, stems, and leaves were opposite. PPO had the highest activity in roots and the lowest activity in leaves, while PAL activity was highest in leaves but lowest in roots. In addition, the contents of soluble protein (SP) and soluble sugar (SS) were measured in the alfalfa plants in the two treatment groups. SP increased significantly in roots but decreased in stems and leaves of the Cp2 group compared with the CK group, while SS content was reduced in the roots but increased in stems and leaves of the Cp2 group compared with the CK group. The changes in SP and SS contents exhibited an opposite trend in the different treatment groups. However, the content of SP showed the same trend as SS content in different tissues of alfalfa, which was a successive significant decrease from leaves to stems and then roots (Figures 9H,I).

### Principal Component Analysis

**FIGURE 10:**
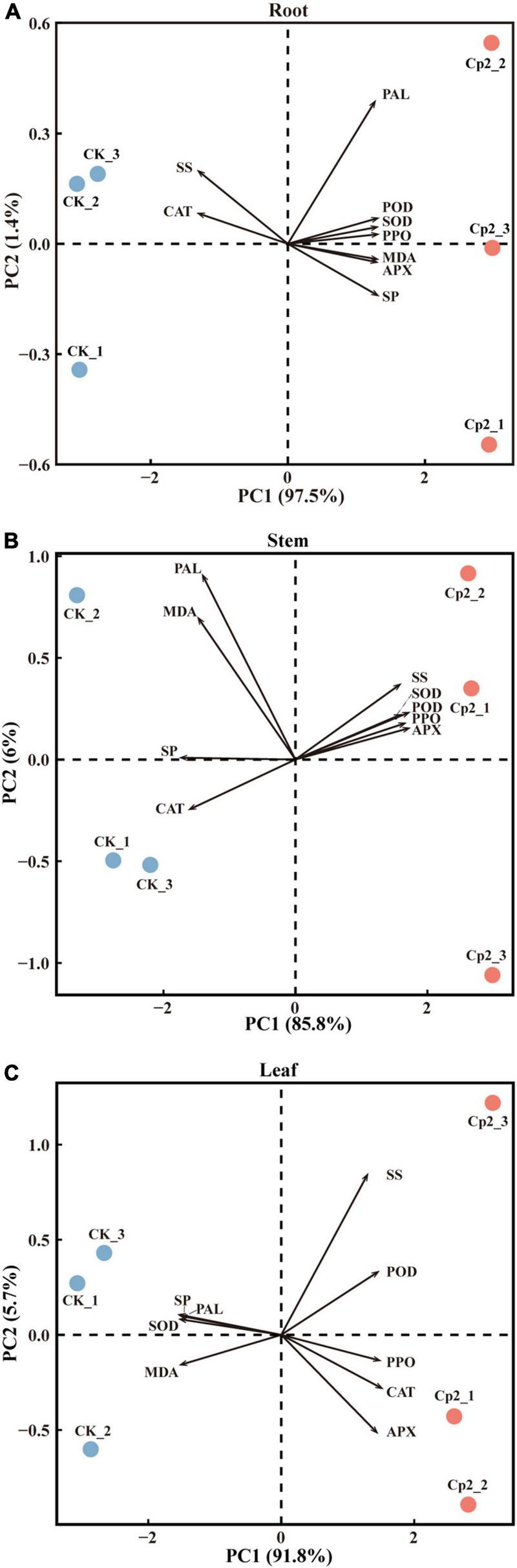
Principal component analysis of physiological indicators of alfalfa inoculated with *E. persicina* Cp2. **(A–C)** Results of physiological indicators of leaf, stem, and root. The percent total variance is shown for PC1 and PC2 in parenthesis in the axis. PCA, principal component analysis; PC1, principal component 1; PC2, principal component 2.

To further screen the physio-biochemical indicators that responded most significantly to the Cp2 treatment, data of the root, stem, and leaf were subjected to PCA. The first principal components (PC1) in the root, stem, and leaf tissue explained 97.5, 85.8, and 91.8%, respectively, of the total variance (Figures 10A–C). The CK and Cp2 samples were well separated on the PC1, therefore the difference of physio-biochemical indicators between the Cp2 and CK groups can be explained by the PC1.

Next, the loadings and contributions associated with the PC1 were analyzed, and nine physio-biochemical indicators were ranked by contributions. Subsequently, the contribution data were divided into three groups by quartile method, namely unimportant indicators (≥0% and <25%), important indicators (≥25% and <75%), and extremely important indicators (≥75% and ≤100%) (Supplementary Table 4). SOD, POD, and SP were extremely important in roots; SP, APX, and POD were extremely important in stems; and SP, SOD, and MDA were extremely important in leaves.

## Discussion

### Inhibition of Alfalfa Roots by *E. persicina* Cp2 May Affect the Establishment of Artificial Grassland

In this study, growth bottles were used as an experimental device to study infection of alfalfa, and deleterious effects of *E. persicina* Cp2 isolated from alfalfa seeds was demonstrated by treating alfalfa roots with bacterial suspensions. Inhibition of alfalfa root growth is an important aspect of the effects induced by *E. persicina* Cp2. We were interested to determine if the inhibition of root growth results from osmotic stress. Our results showed that the water potential of Cp2 suspension was lower than that of CK. But the water potential of root was still lower than that of suspension in the Cp2 group. This suggests that in response to decline of ambient water potential in Cp2 group, alfalfa plants accumulate the osmotic regulatory substances to reduce the water potential of root cells. Usually, soluble sugar and soluble protein act as osmoregulatory substances (Ozturk et al., 2021). In our study, we found that the soluble protein content was significantly increased in roots. This indicates that the soluble protein may be an essential osmotic regulatory substance to reduce alfalfa root water potential and maintain normal water absorption. Sucrose is the major soluble sugar (also including glucose, maltose, and trehalose) in plant (Hershkovitz et al., 1991; Lu et al., 2009). Under abiotic stress, such as drought stress, the soluble sugar content in the alfalfa roots was generally increased (Zhang and Shi, 2018). However, in this research, we found that the root soluble sugar was significantly decreased. We speculate that Cp2 strains may consume root soluble sugar as a carbon source, because in our previous study we reported that *E. persicina* strain Cp2 can utilize various carbon sources, such as sucrose, glucose, maltose, and trehalose (Zhang, 2013). In short, our results showed that osmotic stress was not occurring, so the reasons for this observation may be that the secondary metabolites produced by strain Cp2 have a major adverse influence on root growth of alfalfa during plant-bacteria interactions.

Indole-3-acetic acid (IAA) has been suggested as a prime trigger and regulator for root initiation and growth (Hartmann et al., 1990; Multani et al., 2003; Scarpella et al., 2006), thus IAA might be an essential regulator of root growth of alfalfa. Most plant-associated bacteria can produce IAA (Scott et al., 2013; Finkel et al., 2020) and there are a few reports of IAA production by *E. persicina*. For example, *E. persicina* 2-5b isolated from *Chelidonium majus* L. collected in Poland produced IAA while growing in LB medium (Goryluk-Salmonowicz et al., 2018), and Liang et al. (2021) reported that *E. persicina* isolated from *Elymus nutans* also exhibited IAA production ability. More importantly, IAA biosynthesis gene, *iaaH*, was discovered in the *E. persicina* Cp2 genome (Okal et al., 1999). So, IAA produced by the Cp2 suspensions in the current study could potentially be detrimental to the alfalfa in the study. Bacterial IAA at low levels is usually implicated in promoting root elongation, but a high level of IAA can inhibit root growth and even cause plant death (Gravel et al., 2007; Barth et al., 2010). In the current study, strain Cp2 might have produced a high concentration of bacterial IAA in the 200 mL suspensions in the growth bottle, and this then inhibited growth of the alfalfa roots. Furthermore, we re-isolated strains Cp2 from the roots at day 21, the destruction of cortical cells and root hairs by Cp2 invasion is also likely to have contributed to impaired alfalfa root growth (Nehl et al., 1997). The detailed mechanism of alfalfa root inhibition by *E. persicina* Cp2 requires further investigation in the future.

It is anticipated that alfalfa root shortening by *E. persicina* Cp2 will have a major impact on at least two broad areas of alfalfa production. First, root shortening can influence normal nutrient absorption and will then affect healthy growth of the alfalfa plants. Studies have shown that root length influences nutrient uptake of plants and that there is a relationship between greater root growth and higher yield (Silberbush and Barber, 1983). The second area is that shorter roots do not penetrate the moist subsoil smoothly, which is detrimental to the survival of perennial alfalfa (Haling et al., 2010). Perennial alfalfa is one of the important forages for sustainable artificial grassland, which is widely planted in arid and semi-arid regions (Huang et al., 2018). However, the two effects outlined above suggest that inhibition of root growth by *E. persicina* Cp2 may lead to the death of alfalfa plants in the arid zone. Therefore, to address this potential problem, further studies on the mechanism of root shortening by *E. persicina* Cp2 will be necessary.

### *Erwinia persicina* Invade From the Root and Spread Upward to the Leaf to Cause Disease

Symptoms caused by *E. persicina* Cp2 on the leaves of alfalfa were also examined in the current study. From day 10 after Cp2 inoculation, spots were observed on the leaves of some plants in the growth bottle. Subsequently, more alfalfa plants developed leaf spots and chlorosis symptoms, and the leaves of most plants were necrotic at day 21. These symptoms could be attributed to *E. persicina* Cp2 because the strains Cp2 were re-isolated from these leaves at day 21. Leaf chlorosis is known to be associated with inhibition of chlorophyll biosynthesis in leaf cells. In this research, the decrease in chlorophyll contents (ChlT, Chla, Chlb, and Car) and net photosynthesis (Pn) were observed. We infer that this relates to the iron contents in leaf, because the largest number of virulence factors belongs to the iron uptake system (Figure 3). And iron deficiency can cause great decrease of chlorophyll content, then lead to reduction of photosynthetic rate and therefore a decreased in biomass (Larbi et al., 2006). This is consistent with our results. Leaf disease appears to be a characteristic of *E. persicina* as there are several reports concerning leaf disease induced by *E. persicina*. For example, leaf spot of *Phaseolus vulgaris* and leaf chlorosis of *Pisum sativum* caused by *E. persicina* were observed in southeastern Spain (González et al., 2005, 2007), while leaf disease of cucumber, tomato, melon, spinach, and *Cucurbita pepo* have also been recorded by *E. persicina* (Diánez et al., 2009; Liu et al., 2020; Li et al., 2021). These studies demonstrate that *E. persicina* can cause leaf disease in species of Leguminosae, Cucurbitaceae, Chenopodioideae, and Solanaceae. The alfalfa plant in the current study belongs to the family Leguminosae, and the leaf symptoms observed in this study are similar to those reported in the literature.

In the current study, strains Cp2 were re-isolated from roots, stems, and leaves at day 21. Based on this, we demonstrate that strain Cp2 can invades alfalfa from the root, then transmits vertically up the stem, and finally colonizes the leaves and causes disease. This phenomenon is congruent with the survival strategy of pathogens, whereby bacterial cells move toward regions with more abundant resources or away from noxious regions to reach their preferred niches for colonization (Josenhans and Suerbaum, 2002; Wadhwa and Berg, 2022). For instance, *Ralstonia solanacearum* also enters host plants through the roots, then moves up into the stem and colonizes the xylem tissue (Schell, 2000). However, not all pathogens can migrate freely in their host; the free transfer or movement depends on the motility of the pathogen. The motility of *E. persicina* is well characterized (Gálvez et al., 2015; Yan et al., 2019), and this ability is very beneficial for the infection by the *E. persicina* because bacteria can colonize any suitable environment through motility no matter where they invade the plant host (Kobayashi et al., 2016). During colonization, bacterial cells are known to respond to the environment and move by a process called “chemotaxis” (Wadhams and Armitage, 2004). Therefore, there may be a substance or a class of substances in alfalfa leaves that attracts *E. persicina* to approach. This theory requires further investigation in future studies.

### *Erwinia persicina* Mainly Affects the Antioxidant Enzymes and Soluble Proteins in Alfalfa

In the study of plant-bacteria interactions, researchers generally investigate changes in host physiology, in particular the effect of bacteria on the antioxidant system of host. However, unlike most previous studies, the changes in physio-biochemical indicators of the host root, stem, and leaf were investigated in the current study. Cp2 treatment exhibited a significant effect on antioxidant-associated enzymes of alfalfa, but the indicators of major changes in roots, stems, and leaves were different. The activities of SOD and POD enzymes increased significantly in roots, APX and POD increased significantly in stems. However, the activity of SOD changed most significantly in the leaves and was decreased in this plant part. Otherwise, the activity of CAT was decreased in roots and stems while was increased in leaves. SOD, POD, APX, and CAT enzymes play essential roles in removing reactive oxygen species (ROS) molecules in plants (Nanda et al., 2010). SOD is a key superoxide (O_2_^–^) scavenger, CAT and APX assist the plant in scavenging excessive H_2_O_2_ (De Gara et al., 2003). In this study, the change trend of SOD activity was opposite to that of CAT activity in roots, stems, and leaves. The increased SOD activity in roots and stems may suggest increased O_2_^–^ generation due to strains Cp2, because striking increase of O_2_^–^ in plants has been demonstrated to occur in the early response to plant-pathogen attack (Mehdy, 1994). Kono and Fridovich (1982) reported CAT was inhibited by O_2_^–^, this suggests that the decrease of CAT activity in roots and stems was due to a large amount of O_2_^–^ accumulated. However, H_2_O_2_ can be formed due to O_2_^–^ disproportionation (Sun et al., 2018). In our study, the APX and POD activities were significantly increased in roots and stems, we learned that they are the key enzymes responsible for H_2_O_2_ scavenging (De Gara et al., 2003; Wang et al., 2016). In leaves, the decreased SOD activity may suggest that O_2_^–^ levels were decreased. So the inhibitory effects of O_2_^–^ on CAT activity was decreased, which is consistent with the increased CAT activity we observed. And increased CAT, APX, and POD activities in leaves indicates that considerable accumulation of H_2_O_2_ might be induced by strains Cp2. H_2_O_2_ is the most stable of the ROS and higher amounts of H_2_O_2_ can lead to programmed cell death (PCD) or cell necrosis (Quan et al., 2008; Koshiba et al., 2009). This may be the cause for leaf spot observed in this study. MDA was documented as index of lipid peroxidation, and the content of MDA was an indicator of free radical levels (Yue et al., 2015). Our results showed that the change trend of MDA content was consistent with that of SOD activity. This may be due to the content of MDA is mainly affected by the level of O_2_^–^.

In addition to these findings, another important result was obtained in the current study. PAL is one of the most extensively studied enzymes in the response pathways of plant biotic stress (MacDonald and D’Cunha, 2007). PAL is mainly involved in phenylpropanoid metabolism leading to the production of defensive substances (lignins, coumarins, and flavonoids) in plants (Chandrasekaran and Chun, 2016). We speculated that the increase in PAL activity in roots is connected to lignin biosynthesis of alfalfa roots. However, the PAL activity was decreased in stems and leaves in our study, this indicates that PAL does not appear to play an important role in the resistance of alfalfa plants against strains Cp2. Furthermore, PCA revealed that the changes of SP in roots, stems, and leaves were very significant after Cp2 treatment, and showed that SP increased significantly in roots and decreased in stems and leaves. An interesting result was that contents of SS and SP had the exact opposite trends in roots, stems, and leaves. As we discussed before, the SS contents decreased in alfalfa roots, most likely because these sugars are the energy source for a large number of strains in Cp2 suspension. SS consumption might be reduced in stems and leaves because the strains Cp2 in stems and leaves were less than suspension, so the SS contents was increased to maintain the osmotic equilibrium. We speculated that the synthesis of soluble protein in stems and leaves might be blocked during bacterial infection. The physiological response of alfalfa plants to bacterial infection are complex, the detailed mechanism needs to be further studied in the future.

## Data Availability Statement

The datasets presented in this study can be found in online repositories. The names of the repository/repositories and accession number(s) can be found in the article/Supplementary Material.

## Author Contributions

BY, ZZ, and SS designed the experiments and contributed to the writing and revision of the manuscript. BY performed the experiments, being assisted by RH, analyzed the data, and wrote the manuscript. All authors approved the final version of the manuscript.

## Conflict of Interest

The authors declare that the research was conducted in the absence of any commercial or financial relationships that could be construed as a potential conflict of interest.

## Publisher’s Note

All claims expressed in this article are solely those of the authors and do not necessarily represent those of their affiliated organizations, or those of the publisher, the editors and the reviewers. Any product that may be evaluated in this article, or claim that may be made by its manufacturer, is not guaranteed or endorsed by the publisher.

## Footnotes

1
http://jspecies.ribohost.com/jspeciesws/#analyse

2
https://www.ncbi.nlm.nih.gov/data-hub/taxonomy/551/

3
https://www.omicshare.com/tools/home/report/reportvenn.html
