# Supplementary material for: Seed-Borne Erwinia persicina Affects the Growth and Physiology of Alfalfa (Medicago sativa L.)
Source: Front Microbiol. 2022 May 26;13:891188. doi: 10.3389/fmicb.2022.891188 (PMC9178255; doi:10.3389/fmicb.2022.891188)
Supplement: Supplementary file 1 [file Table_1.DOCX]

| **TABLE S1 \|** Prediction of virulence genes of *E. persicina* strain Cp2 in VFDB | | | | |
| --- | --- | --- | --- | --- |
| **Gene ID** | **Location** | **VFDB ID** | **Virulence factors** | **Categories** |
| gene1445 | Chr | VFG001269(gi:33591935) | Cya | Toxin |
| gene2125 | Chr | VFG001269(gi:33591935) | Cya | Toxin |
| gene2185 | Chr | VFG001269(gi:33591935) | Cya | Toxin |
| gene2240 | Chr | VFG001269(gi:33591935) | Cya | Toxin |
| gene2979 | Chr | VFG001269(gi:33591935) | Cya | Toxin |
| gene3412 | Chr | VFG001269(gi:33591935) | Cya | Toxin |
| gene0035 | Chr | VFG001867(gi:52843161) | SodB | Stress protein |
| gene1003 | Chr | VFG000079(gi:16802278) | ClpC | Stress protein |
| gene1057 | Chr | VFG037118(gi:15676638) | RecN | Stress protein |
| gene1625 | Chr | VFG001861(gi:52840449) | KatAB | Stress protein |
| gene2549 | Chr | VFG037028(gi:15676142) | KatA | Stress protein |
| gene2985 | Chr | VFG000079(gi:16802278) | ClpC | Stress protein |
| gene3398 | Chr | VFG037082(gi:15676491) | MntABC | Stress protein |
| gene3399 | Chr | VFG037064(gi:15676492) | MntABC | Stress protein |
| gene3400 | Chr | VFG037046(gi:15676493) | MntABC | Stress protein |
| gene3428 | Chr | VFG000077(gi:16804506) | ClpP | Stress protein |
| gene3430 | Chr | VFG000077(gi:16804506) | ClpP | Stress protein |
| p_gene0115 | p | VFG037100(gi:15675984) | MsrAB | Stress protein |
| gene0670 | Chr | VFG041911(gi:28901187) | T3SS2 | Secretion system |
| gene0793 | Chr | VFG045607(gi:52843130) | Dot/Icm | Secretion system |
| gene1316 | Chr | VFG002078(gi:15600881) | T6SS | Secretion system |
| gene1760 | Chr | VFG002059(gi:15595271) | HSI-I | Secretion system |
| gene2265 | Chr | VFG002085(gi:15600888) | T6SS | Secretion system |
| gene2581 | Chr | VFG002064(gi:15595276) | HSI-I | Secretion system |
| gene2582 | Chr | VFG002063(gi:15595275) | HSI-I | Secretion system |
| gene2585 | Chr | VFG002061(gi:15595273) | HSI-I | Secretion system |
| gene2732 | Chr | VFG002059(gi:15595271) | HSI-I | Secretion system |
| gene2953 | Chr | VFG045590(gi:52842834) | Dot/Icm | Secretion system |
| gene3985 | Chr | VFG010515(gi:52841406) | Dot/Icm | Secretion system |
| gene4023 | Chr | VFG002078(gi:15600881) | T6SS | Secretion system |
| gene4038 | Chr | VFG002084(gi:15600887) | T6SS | Secretion system |
| gene4043 | Chr | VFG002093(gi:15600879) | T6SS | Secretion system |
| gene4046 | Chr | VFG002064(gi:15595276) | HSI-I | Secretion system |
| gene4488 | Chr | VFG000390(gi:16082728) | TTSS | Secretion system |
| gene0487 | Chr | VFG001386(gi:15607897) | PhoP | Regulation |
| gene0832 | Chr | VFG001386(gi:15607897) | PhoP | Regulation |
| gene0906 | Chr | VFG001826(gi:15609720) | RelA | Regulation |
| gene1376 | Chr | VFG001386(gi:15607897) | PhoP | Regulation |
| gene3190 | Chr | VFG001386(gi:15607897) | PhoP | Regulation |
| gene3473 | Chr | VFG001386(gi:15607897) | PhoP | Regulation |
| gene4427 | Chr | VFG001826(gi:15609720) | RelA | Regulation |
| gene4469 | Chr | VFG001386(gi:15607897) | PhoP | Regulation |
| gene0036 | Chr | VFG001206(gi:15677997) | FbpABC | Iron uptake system |
| gene0078 | Chr | VFG000344(gi:16272073) | HitABC | Iron uptake system |
| gene0091 | Chr | VFG000344(gi:16272073) | HitABC | Iron uptake system |
| gene0214 | Chr | VFG000163(gi:15597594) | Pyoverdine | Iron uptake system |
| gene0225 | Chr | VFG001206(gi:15677997) | FbpABC | Iron uptake system |
| gene0249 | Chr | VFG000344(gi:16272073) | HitABC | Iron uptake system |
| gene0260 | Chr | VFG000344(gi:16272073) | HitABC | Iron uptake system |
| gene0264 | Chr | VFG001206(gi:15677997) | FbpABC | Iron uptake system |
| gene0296 | Chr | VFG001859(gi:52842863) | FeoAB | Iron uptake system |
| gene0297 | Chr | VFG001858(gi:52842864) | FeoAB | Iron uptake system |
| gene0336 | Chr | VFG001206(gi:15677997) | FbpABC | Iron uptake system |
| gene0405 | Chr | VFG000344(gi:16272073) | HitABC | Iron uptake system |
| gene0461 | Chr | VFG000344(gi:16272073) | HitABC | Iron uptake system |
| gene0467 | Chr | VFG001206(gi:15677997) | FbpABC | Iron uptake system |
| gene0687 | Chr | VFG001206(gi:15677997) | FbpABC | Iron uptake system |
| gene0864 | Chr | VFG001820(gi:15609516) | Mycobactin | Iron uptake system |
| gene0917 | Chr | VFG001206(gi:15677997) | FbpABC | Iron uptake system |
| gene1022 | Chr | VFG001206(gi:15677997) | FbpABC | Iron uptake system |
| gene1149 | Chr | VFG000344(gi:16272073) | HitABC | Iron uptake system |
| gene1188 | Chr | VFG001206(gi:15677997) | FbpABC | Iron uptake system |
| gene1218 | Chr | VFG001206(gi:15677997) | FbpABC | Iron uptake system |
| gene1323 | Chr | VFG001856(gi:52841094) | CcmC | Iron uptake system |
| gene1362 | Chr | VFG001206(gi:15677997) | FbpABC | Iron uptake system |
| gene1391 | Chr | VFG001206(gi:15677997) | FbpABC | Iron uptake system |
| gene1451 | Chr | VFG000160(gi:15597593) | Pyoverdine | Iron uptake system |
| gene1452 | Chr | VFG001206(gi:15677997) | FbpABC | Iron uptake system |
| gene1474 | Chr | VFG000344(gi:16272073) | HitABC | Iron uptake system |
| gene1503 | Chr | VFG000344(gi:16272073) | HitABC | Iron uptake system |
| gene1614 | Chr | VFG001206(gi:15677997) | FbpABC | Iron uptake system |
| gene1620 | Chr | VFG001206(gi:15677997) | FbpABC | Iron uptake system |
| gene1641 | Chr | VFG001818(gi:15609514) | Mycobactin | Iron uptake system |
| gene1643 | Chr | VFG000163(gi:15597594) | Pyoverdine | Iron uptake system |
| gene1645 | Chr | VFG000163(gi:15597594) | Pyoverdine | Iron uptake system |
| gene1676 | Chr | VFG000344(gi:16272073) | HitABC | Iron uptake system |
| gene1684 | Chr | VFG000359(gi:16122155) | Yersiniabactin | Iron uptake system |
| gene1685 | Chr | VFG000169(gi:15599425) | Pyochelin | Iron uptake system |
| gene1689 | Chr | VFG000362(gi:16122158) | Yersiniabactin | Iron uptake system |
| gene1718 | Chr | VFG000344(gi:16272073) | HitABC | Iron uptake system |
| gene1731 | Chr | VFG000344(gi:16272073) | HitABC | Iron uptake system |
| gene1751 | Chr | VFG001206(gi:15677997) | FbpABC | Iron uptake system |
| gene1752 | Chr | VFG001266(gi:15599418) | Pyochelin | Iron uptake system |
| gene1803 | Chr | VFG000344(gi:16272073) | HitABC | Iron uptake system |
| gene1852 | Chr | VFG001206(gi:15677997) | FbpABC | Iron uptake system |
| gene2184 | Chr | VFG001206(gi:15677997) | FbpABC | Iron uptake system |
| gene2204 | Chr | VFG001206(gi:15677997) | FbpABC | Iron uptake system |
| gene2315 | Chr | VFG000163(gi:15597594) | Pyoverdine | Iron uptake system |
| gene2323 | Chr | VFG000344(gi:16272073) | HitABC | Iron uptake system |
| gene2339 | Chr | VFG000619(gi:56480401) | Aerobactin | Iron uptake system |
| gene2346 | Chr | VFG000268(gi:15793475) | HpuAB | Iron uptake system |
| gene2371 | Chr | VFG001206(gi:15677997) | FbpABC | Iron uptake system |
| gene2444 | Chr | VFG000366(gi:16122162) | Yersiniabactin | Iron uptake system |
| gene2794 | Chr | VFG000362(gi:16122158) | Yersiniabactin | Iron uptake system |
| gene2939 | Chr | VFG001206(gi:15677997) | FbpABC | Iron uptake system |
| gene3006 | Chr | VFG000344(gi:16272073) | HitABC | Iron uptake system |
| gene3029 | Chr | VFG000344(gi:16272073) | HitABC | Iron uptake system |
| gene3050 | Chr | VFG000344(gi:16272073) | HitABC | Iron uptake system |
| gene3094 | Chr | VFG001206(gi:15677997) | FbpABC | Iron uptake system |
| gene3095 | Chr | VFG001206(gi:15677997) | FbpABC | Iron uptake system |
| gene3228 | Chr | VFG000344(gi:16272073) | HitABC | Iron uptake system |
| gene3285 | Chr | VFG001206(gi:15677997) | FbpABC | Iron uptake system |
| gene3349 | Chr | VFG001267(gi:15599419) | Pyochelin | Iron uptake system |
| gene3441 | Chr | VFG045330(gi:52841554) | Legiobactin | Iron uptake system |
| gene3509 | Chr | VFG000344(gi:16272073) | HitABC | Iron uptake system |
| gene3559 | Chr | VFG001206(gi:15677997) | FbpABC | Iron uptake system |
| gene3591 | Chr | VFG000344(gi:16272073) | HitABC | Iron uptake system |
| gene3737 | Chr | VFG000344(gi:16272073) | HitABC | Iron uptake system |
| gene3797 | Chr | VFG000344(gi:16272073) | HitABC | Iron uptake system |
| gene3841 | Chr | VFG000341(gi:16272221) | HxuABC | Iron uptake system |
| gene4075 | Chr | VFG001206(gi:15677997) | FbpABC | Iron uptake system |
| gene4101 | Chr | VFG001817(gi:15609521) | Mycobactin | Iron uptake system |
| gene4499 | Chr | VFG000344(gi:16272073) | HitABC | Iron uptake system |
| p_gene0110 | p | VFG000344(gi:16272073) | HitABC | Iron uptake system |
| p_gene0139 | p | VFG001206(gi:15677997) | FbpABC | Iron uptake system |
| gene0251 | Chr | VFG002319(gi:123442749) | Flagella | Invasion |
| gene0611 | Chr | VFG002319(gi:123442749) | Flagella | Invasion |
| gene0769 | Chr | VFG002529(gi:53720914) | Flagella | Invasion |
| gene0947 | Chr | VFG002319(gi:123442749) | Flagella | Invasion |
| gene1794 | Chr | VFG002529(gi:53720914) | Flagella | Invasion |
| gene1853 | Chr | VFG002157(gi:16803887) | LpeA | Invasion |
| gene1875 | Chr | VFG002326(gi:123442756) | Flagella | Invasion |
| gene1876 | Chr | VFG002325(gi:123442755) | Flagella | Invasion |
| gene1877 | Chr | VFG002324(gi:123442754) | Flagella | Invasion |
| gene1878 | Chr | VFG002322(gi:123442752) | Flagella | Invasion |
| gene1888 | Chr | VFG002319(gi:123442749) | Flagella | Invasion |
| gene1889 | Chr | VFG002318(gi:123442748) | Flagella | Invasion |
| gene1938 | Chr | VFG002359(gi:123442807) | Flagella | Invasion |
| gene1939 | Chr | VFG002358(gi:123442806) | Flagella | Invasion |
| gene1952 | Chr | VFG002357(gi:123442794) | Flagella | Invasion |
| gene1953 | Chr | VFG002356(gi:123442793) | Flagella | Invasion |
| gene2072 | Chr | VFG011850(gi:15791455) | Flagella | Invasion |
| gene2754 | Chr | VFG002327(gi:123442763) | Flagella | Invasion |
| gene2755 | Chr | VFG002328(gi:123442764) | Flagella | Invasion |
| gene2756 | Chr | VFG002329(gi:123442765) | Flagella | Invasion |
| gene2757 | Chr | VFG002330(gi:123442766) | Flagella | Invasion |
| gene2758 | Chr | VFG002331(gi:123442767) | Flagella | Invasion |
| gene2759 | Chr | VFG002332(gi:123442768) | Flagella | Invasion |
| gene2760 | Chr | VFG002333(gi:123442769) | Flagella | Invasion |
| gene2761 | Chr | VFG002334(gi:123442770) | Flagella | Invasion |
| gene2762 | Chr | VFG002335(gi:123442771) | Flagella | Invasion |
| gene2763 | Chr | VFG002336(gi:123442772) | Flagella | Invasion |
| gene2764 | Chr | VFG002337(gi:123442773) | Flagella | Invasion |
| gene2765 | Chr | VFG002338(gi:123442774) | Flagella | Invasion |
| gene2766 | Chr | VFG002339(gi:123442775) | Flagella | Invasion |
| gene2767 | Chr | VFG002340(gi:123442776) | Flagella | Invasion |
| gene2807 | Chr | VFG002341(gi:123442777) | Flagella | Invasion |
| gene2808 | Chr | VFG002342(gi:123442778) | Flagella | Invasion |
| gene2809 | Chr | VFG002343(gi:123442779) | Flagella | Invasion |
| gene2810 | Chr | VFG002344(gi:123442780) | Flagella | Invasion |
| gene2811 | Chr | VFG002345(gi:123442781) | Flagella | Invasion |
| gene2812 | Chr | VFG002513(gi:53717916) | Flagella | Invasion |
| gene2813 | Chr | VFG002347(gi:123442783) | Flagella | Invasion |
| gene2814 | Chr | VFG002348(gi:123442784) | Flagella | Invasion |
| gene2815 | Chr | VFG002349(gi:123442785) | Flagella | Invasion |
| gene2816 | Chr | VFG002350(gi:123442786) | Flagella | Invasion |
| gene2817 | Chr | VFG002351(gi:123442787) | Flagella | Invasion |
| gene2818 | Chr | VFG002352(gi:123442788) | Flagella | Invasion |
| gene2819 | Chr | VFG002353(gi:123442789) | Flagella | Invasion |
| gene3145 | Chr | VFG002440(gi:53722543) | Bsa T3SS | Invasion |
| gene3321 | Chr | VFG002322(gi:123442752) | Flagella | Invasion |
| gene3944 | Chr | VFG002529(gi:53720914) | Flagella | Invasion |
| gene4486 | Chr | VFG001008(gi:31983538) | TTSS | Invasion |
| gene4186 | Chr | VFG001381(gi:57116734) | Isocitrate lyase | Cellular metabolism |
| gene0156 | Chr | VFG000116(gi:15600676) | Alginate | Antiphagocytosis |
| gene0755 | Chr | VFG000120(gi:15600455) | Alginate | Antiphagocytosis |
| gene0948 | Chr | VFG000119(gi:15600454) | Alginate | Antiphagocytosis |
| gene1068 | Chr | VFG000121(gi:15595959) | Alginate | Antiphagocytosis |
| gene1254 | Chr | VFG000120(gi:15600455) | Alginate | Antiphagocytosis |
| gene1555 | Chr | VFG001298(gi:21281854) | Capsule | Antiphagocytosis |
| gene1695 | Chr | VFG002548(gi:53720398) | Capsule I | Antiphagocytosis |
| gene1923 | Chr | VFG001341(gi:22537322) | Capsule | Antiphagocytosis |
| gene2113 | Chr | VFG000122(gi:15598736) | Alginate | Antiphagocytosis |
| gene2197 | Chr | VFG000116(gi:15600676) | Alginate | Antiphagocytosis |
| gene2634 | Chr | VFG002546(gi:53720396) | Capsule I | Antiphagocytosis |
| gene3069 | Chr | VFG002550(gi:53720400) | Capsule I | Antiphagocytosis |
| gene3124 | Chr | VFG002546(gi:53720396) | Capsule I | Antiphagocytosis |
| gene3590 | Chr | VFG002552(gi:53720402) | Capsule I | Antiphagocytosis |
| gene3618 | Chr | VFG002189(gi:29376986) | Capsule | Antiphagocytosis |
| gene3619 | Chr | VFG002190(gi:29376987) | Capsule | Antiphagocytosis |
| gene3849 | Chr | VFG002548(gi:53720398) | Capsule I | Antiphagocytosis |
| gene4203 | Chr | VFG000118(gi:15600448) | Alginate | Antiphagocytosis |
| gene4289 | Chr | VFG001311(gi:21281867) | Capsule | Antiphagocytosis |
| gene4290 | Chr | VFG001373(gi:15900286) | Capsule | Antiphagocytosis |
| gene4379 | Chr | VFG002546(gi:53720396) | Capsule I | Antiphagocytosis |
| gene4393 | Chr | VFG001341(gi:22537322) | Capsule | Antiphagocytosis |
| p_gene0068 | p | VFG001306(gi:21281862) | Capsule | Antiphagocytosis |
| gene0022 | Chr | VFG000335(gi:16273088) | P5 protein | Adherence |
| gene0125 | Chr | VFG001234(gi:15595613) | Type IV pili | Adherence |
| gene0465 | Chr | VFG001971(gi:15792761) | Capsule | Adherence |
| gene0599 | Chr | VFG045346(gi:37679074) | IlpA | Adherence |
| gene0789 | Chr | VFG000232(gi:15675990) | Type IV pili | Adherence |
| gene0990 | Chr | VFG001931(gi:15792793) | CadF | Adherence |
| gene1194 | Chr | VFG001225(gi:15595605) | Type IV pili | Adherence |
| gene1309 | Chr | VFG001931(gi:15792793) | CadF | Adherence |
| gene1358 | Chr | VFG001971(gi:15792761) | Capsule | Adherence |
| gene1558 | Chr | VFG001947(gi:15792471) | LOS | Adherence |
| gene2042 | Chr | VFG045345(gi:28898385) | MAM7 | Adherence |
| gene2117 | Chr | VFG006717(gi:16803674) | Lap | Adherence |
| gene2190 | Chr | VFG001214(gi:15599743) | Type IV pili | Adherence |
| gene2239 | Chr | VFG045346(gi:37679074) | IlpA | Adherence |
| gene2376 | Chr | VFG001249(gi:15596296) | Flagella | Adherence |
| gene2406 | Chr | VFG001213(gi:15599742) | Type IV pili | Adherence |
| gene2868 | Chr | VFG001971(gi:15792761) | Capsule | Adherence |
| gene3160 | Chr | VFG001931(gi:15792793) | CadF | Adherence |
| gene3283 | Chr | VFG006717(gi:16803674) | Lap | Adherence |
| gene3593 | Chr | VFG045346(gi:37679074) | IlpA | Adherence |
| gene3697 | Chr | VFG000112(gi:15599722) | Type IV pili | Adherence |
| gene3698 | Chr | VFG000113(gi:15599723) | Type IV pili | Adherence |
| gene3704 | Chr | VFG000114(gi:15599724) | Type IV pili | Adherence |
| gene3770 | Chr | VFG001145(gi:535166) | Haemagglutinating pili | Adherence |
| gene3783 | Chr | VFG001226(gi:15595606) | Type IV pili | Adherence |
| gene3984 | Chr | VFG002437(gi:53719320) | BoaB | Adherence |
| gene4009 | Chr | VFG001855(gi:52840925) | Hsp60 | Adherence |
| gene4121 | Chr | VFG001234(gi:15595613) | Type IV pili | Adherence |
| gene4368 | Chr | VFG001225(gi:15595605) | Type IV pili | Adherence |
| gene4383 | Chr | VFG000142(gi:15600204) | LPS | Adherence |
| gene4387 | Chr | VFG000316(gi:15644907) | LPS | Adherence |
| gene4389 | Chr | VFG000142(gi:15600204) | LPS | Adherence |
| gene4390 | Chr | VFG000139(gi:15600203) | LPS | Adherence |
| gene4396 | Chr | VFG000320(gi:15646084) | LPS | Adherence |
| gene4441 | Chr | VFG001214(gi:15599743) | Type IV pili | Adherence |
| p_gene0067 | p | VFG000104(gi:15640857) | ACF | Adherence |
| gene0067 | Chr | VFG005767(gi:22536831) | Beta-hemolysin/cytolysin | - |
| gene0090 | Chr | VFG000925(gi:26246567) | Enterobactin | - |
| gene0119 | Chr | VFG000574(gi:16767047) | MgtBC | - |
| gene0154 | Chr | VFG007023(gi:15641459) | RTX toxin | - |
| gene0178 | Chr | VFG002414(gi:15799998) | ECP | - |
| gene0179 | Chr | VFG002412(gi:15799997) | ECP | - |
| gene0180 | Chr | VFG002415(gi:15799996) | ECP | - |
| gene0185 | Chr | VFG007023(gi:15641459) | RTX toxin | - |
| gene0197 | Chr | VFG043319(gi:52841089) | polar flagella | - |
| gene0217 | Chr | VFG045467(gi:53718898) | CdpA | - |
| gene0259 | Chr | VFG005776(gi:22536834) | Beta-hemolysin/cytolysin | - |
| gene0269 | Chr | VFG005767(gi:22536831) | Beta-hemolysin/cytolysin | - |
| gene0290 | Chr | VFG005788(gi:22536838) | Beta-hemolysin/cytolysin | - |
| gene0301 | Chr | VFG009810(gi:15607898) | PhoP/R | - |
| gene0313 | Chr | VFG010463(gi:52841166) | type IV pili | - |
| gene0325 | Chr | VFG015515(gi:15599409) | Phenazines biosynthesis | - |
| gene0342 | Chr | VFG002176(gi:21693305) | Cytolysin | - |
| gene0397 | Chr | VFG045340(gi:17987019) | RicA | - |
| gene0411 | Chr | VFG013436(gi:30995456) | LOS | - |
| gene0443 | Chr | VFG014984(gi:15599642) | Alginate regulation | - |
| gene0444 | Chr | VFG014950(gi:15595963) | Alginate regulation | - |
| gene0454 | Chr | VFG009810(gi:15607898) | PhoP/R | - |
| gene0482 | Chr | VFG000934(gi:26246575) | Enterobactin | - |
| gene0483 | Chr | VFG000933(gi:26246574) | Enterobactin | - |
| gene0484 | Chr | VFG000932(gi:26246573) | Enterobactin | - |
| gene0485 | Chr | VFG000931(gi:26246572) | Enterobactin | - |
| gene0577 | Chr | VFG036974(gi:15677563) | MtrCDE | - |
| gene0578 | Chr | VFG036992(gi:15677562) | MtrCDE | - |
| gene0581 | Chr | VFG000574(gi:16767047) | MgtBC | - |
| gene0594 | Chr | VFG005776(gi:22536834) | Beta-hemolysin/cytolysin | - |
| gene0607 | Chr | VFG044083(gi:15597454) | pyoverdine | - |
| gene0608 | Chr | VFG043209(gi:123442801) | peritrichous flagella | - |
| gene0622 | Chr | VFG000331(gi:16273426) | LOS | - |
| gene0635 | Chr | VFG044083(gi:15597454) | pyoverdine | - |
| gene0652 | Chr | VFG016046(gi:15597609) | pyoverdine | - |
| gene0665 | Chr | VFG045467(gi:53718898) | CdpA | - |
| gene0716 | Chr | VFG002360(gi:123443278) | O-antigen | - |
| gene0720 | Chr | VFG043366(gi:15644729) | Pse5Ac7Ac | - |
| gene0722 | Chr | VFG045467(gi:53718898) | CdpA | - |
| gene0726 | Chr | VFG000925(gi:26246567) | Enterobactin | - |
| gene0727 | Chr | VFG000922(gi:26250139) | Chu | - |
| gene0728 | Chr | VFG000922(gi:26250139) | Chu | - |
| gene0730 | Chr | VFG000935(gi:26247124) | IroN | - |
| gene0738 | Chr | VFG000935(gi:26247124) | IroN | - |
| gene0739 | Chr | VFG000924(gi:26246571) | Enterobactin | - |
| gene0742 | Chr | VFG000925(gi:26246567) | Enterobactin | - |
| gene0754 | Chr | VFG000925(gi:26246567) | Enterobactin | - |
| gene0763 | Chr | VFG045467(gi:53718898) | CdpA | - |
| gene0786 | Chr | VFG013265(gi:16272217) | LOS | - |
| gene0826 | Chr | VFG005767(gi:22536831) | Beta-hemolysin/cytolysin | - |
| gene0853 | Chr | VFG002197(gi:29375537) | BopD | - |
| gene0857 | Chr | VFG036974(gi:15677563) | MtrCDE | - |
| gene0858 | Chr | VFG036992(gi:15677562) | MtrCDE | - |
| gene0859 | Chr | VFG037010(gi:15677561) | MtrCDE | - |
| gene0861 | Chr | VFG036992(gi:15677562) | MtrCDE | - |
| gene0895 | Chr | VFG044083(gi:15597454) | pyoverdine | - |
| gene0905 | Chr | VFG043394(gi:218561946) | Pse5Ac7Ac, Pse5Ac7Am, Pse8OAc, Pse5Am7AcGlnAc | - |
| gene0911 | Chr | VFG032992(gi:16803331) | OatA | - |
| gene0912 | Chr | VFG043209(gi:123442801) | peritrichous flagella | - |
| gene0915 | Chr | VFG044083(gi:15597454) | pyoverdine | - |
| gene0955 | Chr | VFG000907(gi:26249408) | Hemolysin | - |
| gene1015 | Chr | VFG036956(gi:15676236) | FarAB | - |
| gene1016 | Chr | VFG036938(gi:15676235) | FarAB | - |
| gene1017 | Chr | VFG001713(gi:26249417) | P fimbriae | - |
| gene1060 | Chr | VFG043209(gi:123442801) | peritrichous flagella | - |
| gene1077 | Chr | VFG043390(gi:15646189) | Pse5Ac7Ac | - |
| gene1093 | Chr | VFG043345(gi:52841989) | polar flagella | - |
| gene1143 | Chr | VFG045467(gi:53718898) | CdpA | - |
| gene1163 | Chr | VFG045467(gi:53718898) | CdpA | - |
| gene1177 | Chr | VFG036992(gi:15677562) | MtrCDE | - |
| gene1195 | Chr | VFG009810(gi:15607898) | PhoP/R | - |
| gene1221 | Chr | VFG043346(gi:52841990) | polar flagella | - |
| gene1234 | Chr | VFG044083(gi:15597454) | pyoverdine | - |
| gene1291 | Chr | VFG036938(gi:15676235) | FarAB | - |
| gene1302 | Chr | VFG043209(gi:123442801) | peritrichous flagella | - |
| gene1321 | Chr | VFG010862(gi:52841092) | Cytochrome c muturation (ccm) locus | - |
| gene1322 | Chr | VFG010866(gi:52841093) | Cytochrome c muturation (ccm) locus | - |
| gene1325 | Chr | VFG010878(gi:52841096) | Cytochrome c muturation (ccm) locus | - |
| gene1326 | Chr | VFG010882(gi:52841097) | Cytochrome c muturation (ccm) locus | - |
| gene1344 | Chr | VFG045467(gi:53718898) | CdpA | - |
| gene1395 | Chr | VFG016046(gi:15597609) | pyoverdine | - |
| gene1404 | Chr | VFG044083(gi:15597454) | pyoverdine | - |
| gene1435 | Chr | VFG043345(gi:52841989) | polar flagella | - |
| gene1446 | Chr | VFG000908(gi:26249409) | Hemolysin | - |
| gene1493 | Chr | VFG000928(gi:26246568) | Enterobactin | - |
| gene1494 | Chr | VFG000925(gi:26246567) | Enterobactin | - |
| gene1501 | Chr | VFG002197(gi:29375537) | BopD | - |
| gene1513 | Chr | VFG043350(gi:52842010) | polar flagella | - |
| gene1537 | Chr | VFG009810(gi:15607898) | PhoP/R | - |
| gene1539 | Chr | VFG036992(gi:15677562) | MtrCDE | - |
| gene1541 | Chr | VFG036974(gi:15677563) | MtrCDE | - |
| gene1546 | Chr | VFG045467(gi:53718898) | CdpA | - |
| gene1552 | Chr | VFG013365(gi:16272813) | LOS | - |
| gene1559 | Chr | VFG013496(gi:16273585) | LOS | - |
| gene1565 | Chr | VFG013346(gi:16272753) | LOS | - |
| gene1566 | Chr | VFG013286(gi:16272302) | LOS | - |
| gene1567 | Chr | VFG013508(gi:16273603) | LOS | - |
| gene1568 | Chr | VFG013368(gi:30995408) | LOS | - |
| gene1571 | Chr | VFG000033(gi:33591346) | LPS | - |
| gene1635 | Chr | VFG000924(gi:26246571) | Enterobactin | - |
| gene1636 | Chr | VFG044165(gi:26246570) | enterobactin | - |
| gene1637 | Chr | VFG000926(gi:26246569) | Enterobactin | - |
| gene1638 | Chr | VFG000928(gi:26246568) | Enterobactin | - |
| gene1639 | Chr | VFG000925(gi:26246567) | Enterobactin | - |
| gene1640 | Chr | VFG016041(gi:15597598) | pyoverdine | - |
| gene1642 | Chr | VFG044159(gi:26246563) | enterobactin | - |
| gene1647 | Chr | VFG043319(gi:52841089) | polar flagella | - |
| gene1654 | Chr | VFG044083(gi:15597454) | pyoverdine | - |
| gene1679 | Chr | VFG002197(gi:29375537) | BopD | - |
| gene1691 | Chr | VFG013064(gi:82778670) | Shu | - |
| gene1701 | Chr | VFG044083(gi:15597454) | pyoverdine | - |
| gene1709 | Chr | VFG009810(gi:15607898) | PhoP/R | - |
| gene1721 | Chr | VFG044083(gi:15597454) | pyoverdine | - |
| gene1736 | Chr | VFG016046(gi:15597609) | pyoverdine | - |
| gene1745 | Chr | VFG002538(gi:53721911) | Quorum-sensing | - |
| gene1773 | Chr | VFG007023(gi:15641459) | RTX toxin | - |
| gene1787 | Chr | VFG000925(gi:26246567) | Enterobactin | - |
| gene1796 | Chr | VFG002197(gi:29375537) | BopD | - |
| gene1811 | Chr | VFG002197(gi:29375537) | BopD | - |
| gene1813 | Chr | VFG043209(gi:123442801) | peritrichous flagella | - |
| gene1816 | Chr | VFG044083(gi:15597454) | pyoverdine | - |
| gene1828 | Chr | VFG044083(gi:15597454) | pyoverdine | - |
| gene1881 | Chr | VFG000036(gi:33591349) | LPS | - |
| gene1940 | Chr | VFG043213(gi:123442805) | peritrichous flagella | - |
| gene1941 | Chr | VFG043212(gi:123442804) | peritrichous flagella | - |
| gene1942 | Chr | VFG043211(gi:123442803) | peritrichous flagella | - |
| gene1943 | Chr | VFG043210(gi:123442802) | peritrichous flagella | - |
| gene1944 | Chr | VFG043209(gi:123442801) | peritrichous flagella | - |
| gene1945 | Chr | VFG043209(gi:123442801) | peritrichous flagella | - |
| gene1946 | Chr | VFG043209(gi:123442801) | peritrichous flagella | - |
| gene1947 | Chr | VFG043209(gi:123442801) | peritrichous flagella | - |
| gene1948 | Chr | VFG043208(gi:123442799) | peritrichous flagella | - |
| gene1949 | Chr | VFG043207(gi:123442798) | peritrichous flagella | - |
| gene1950 | Chr | VFG043206(gi:123442797) | peritrichous flagella | - |
| gene1951 | Chr | VFG043205(gi:123442796) | peritrichous flagella | - |
| gene1976 | Chr | VFG044172(gi:26250140) | Chu | - |
| gene1979 | Chr | VFG013254(gi:16272163) | LOS | - |
| gene2060 | Chr | VFG045467(gi:53718898) | CdpA | - |
| gene2069 | Chr | VFG016058(gi:15597620) | pyoverdine | - |
| gene2084 | Chr | VFG013365(gi:16272813) | LOS | - |
| gene2111 | Chr | VFG043386(gi:15645681) | Pse5Ac7Ac | - |
| gene2112 | Chr | VFG013346(gi:16272753) | LOS | - |
| gene2124 | Chr | VFG007023(gi:15641459) | RTX toxin | - |
| gene2173 | Chr | VFG045467(gi:53718898) | CdpA | - |
| gene2274 | Chr | VFG043209(gi:123442801) | peritrichous flagella | - |
| gene2275 | Chr | VFG045467(gi:53718898) | CdpA | - |
| gene2279 | Chr | VFG000907(gi:26249408) | Hemolysin | - |
| gene2286 | Chr | VFG044083(gi:15597454) | pyoverdine | - |
| gene2319 | Chr | VFG002158(gi:16802971) | LplA1 | - |
| gene2332 | Chr | VFG043366(gi:15644729) | Pse5Ac7Ac | - |
| gene2335 | Chr | VFG000925(gi:26246567) | Enterobactin | - |
| gene2336 | Chr | VFG000928(gi:26246568) | Enterobactin | - |
| gene2337 | Chr | VFG000926(gi:26246569) | Enterobactin | - |
| gene2381 | Chr | VFG044083(gi:15597454) | pyoverdine | - |
| gene2389 | Chr | VFG043209(gi:123442801) | peritrichous flagella | - |
| gene2417 | Chr | VFG000575(gi:16767048) | MgtBC | - |
| gene2437 | Chr | VFG002197(gi:29375537) | BopD | - |
| gene2453 | Chr | VFG013070(gi:82778664) | Shu | - |
| gene2487 | Chr | VFG043392(gi:218561919) | Pse5Ac7Ac, Pse5Ac7Am, Pse8OAc, Pse5Am7AcGlnAc | - |
| gene2494 | Chr | VFG002197(gi:29375537) | BopD | - |
| gene2519 | Chr | VFG000916(gi:26250129) | Chu | - |
| gene2520 | Chr | VFG013065(gi:82778668) | Shu | - |
| gene2521 | Chr | VFG000922(gi:26250139) | Chu | - |
| gene2522 | Chr | VFG044172(gi:26250140) | Chu | - |
| gene2526 | Chr | VFG044172(gi:26250140) | Chu | - |
| gene2528 | Chr | VFG000922(gi:26250139) | Chu | - |
| gene2544 | Chr | VFG044083(gi:15597454) | pyoverdine | - |
| gene2561 | Chr | VFG016046(gi:15597609) | pyoverdine | - |
| gene2564 | Chr | VFG002307(gi:16765837) | SinH | - |
| gene2567 | Chr | VFG005767(gi:22536831) | Beta-hemolysin/cytolysin | - |
| gene2570 | Chr | VFG000841(gi:75994495) | Hemolysin | - |
| gene2573 | Chr | VFG005767(gi:22536831) | Beta-hemolysin/cytolysin | - |
| gene2598 | Chr | VFG013465(gi:16273457) | LOS | - |
| gene2627 | Chr | VFG043209(gi:123442801) | peritrichous flagella | - |
| gene2631 | Chr | VFG016058(gi:15597620) | pyoverdine | - |
| gene2656 | Chr | VFG043209(gi:123442801) | peritrichous flagella | - |
| gene2675 | Chr | VFG044083(gi:15597454) | pyoverdine | - |
| gene2744 | Chr | VFG043386(gi:15645681) | Pse5Ac7Ac | - |
| gene2769 | Chr | VFG043209(gi:123442801) | peritrichous flagella | - |
| gene2795 | Chr | VFG011430(gi:17987758) | LPS | - |
| gene2796 | Chr | VFG005767(gi:22536831) | Beta-hemolysin/cytolysin | - |
| gene2836 | Chr | VFG013436(gi:30995456) | LOS | - |
| gene2858 | Chr | VFG036938(gi:15676235) | FarAB | - |
| gene2878 | Chr | VFG002540(gi:53722204) | Quorum-sensing | - |
| gene2900 | Chr | VFG000885(gi:26249424) | P fimbriae | - |
| gene2901 | Chr | VFG000884(gi:26249425) | P fimbriae | - |
| gene2922 | Chr | VFG001443(gi:7188818) | OmpA | - |
| gene2930 | Chr | VFG002176(gi:21693305) | Cytolysin | - |
| gene2955 | Chr | VFG013236(gi:16272032) | LOS | - |
| gene2958 | Chr | VFG013242(gi:16272033) | LOS | - |
| gene2959 | Chr | VFG013248(gi:16272034) | LOS | - |
| gene2980 | Chr | VFG000907(gi:26249408) | Hemolysin | - |
| gene2996 | Chr | VFG005776(gi:22536834) | Beta-hemolysin/cytolysin | - |
| gene3034 | Chr | VFG000925(gi:26246567) | Enterobactin | - |
| gene3037 | Chr | VFG036956(gi:15676236) | FarAB | - |
| gene3038 | Chr | VFG036938(gi:15676235) | FarAB | - |
| gene3043 | Chr | VFG000442(gi:17233474) | Rck | - |
| gene3103 | Chr | VFG005767(gi:22536831) | Beta-hemolysin/cytolysin | - |
| gene3112 | Chr | VFG043209(gi:123442801) | peritrichous flagella | - |
| gene3121 | Chr | VFG005776(gi:22536834) | Beta-hemolysin/cytolysin | - |
| gene3133 | Chr | VFG012509(gi:26247127) | Salmochelin | - |
| gene3138 | Chr | VFG009579(gi:15608489) | mycobactin | - |
| gene3203 | Chr | VFG044083(gi:15597454) | pyoverdine | - |
| gene3270 | Chr | VFG010862(gi:52841092) | Cytochrome c muturation (ccm) locus | - |
| gene3275 | Chr | VFG002230(gi:17987701) | LPS | - |
| gene3276 | Chr | VFG000670(gi:24111748) | LPS | - |
| gene3277 | Chr | VFG000033(gi:33591346) | LPS | - |
| gene3284 | Chr | VFG043209(gi:123442801) | peritrichous flagella | - |
| gene3298 | Chr | VFG005767(gi:22536831) | Beta-hemolysin/cytolysin | - |
| gene3302 | Chr | VFG011430(gi:17987758) | LPS | - |
| gene3331 | Chr | VFG000885(gi:26249424) | P fimbriae | - |
| gene3332 | Chr | VFG000884(gi:26249425) | P fimbriae | - |
| gene3334 | Chr | VFG000882(gi:26249427) | P fimbriae | - |
| gene3345 | Chr | VFG013321(gi:16272676) | LOS | - |
| gene3366 | Chr | VFG044083(gi:15597454) | pyoverdine | - |
| gene3386 | Chr | VFG000442(gi:17233474) | Rck | - |
| gene3393 | Chr | VFG036974(gi:15677563) | MtrCDE | - |
| gene3394 | Chr | VFG036992(gi:15677562) | MtrCDE | - |
| gene3411 | Chr | VFG013248(gi:16272034) | LOS | - |
| gene3472 | Chr | VFG009810(gi:15607898) | PhoP/R | - |
| gene3508 | Chr | VFG000841(gi:75994495) | Hemolysin | - |
| gene3545 | Chr | VFG013418(gi:16273103) | LOS | - |
| gene3611 | Chr | VFG013384(gi:16272991) | LOS | - |
| gene3612 | Chr | VFG013390(gi:16272992) | LOS | - |
| gene3613 | Chr | VFG011399(gi:17987115) | LPS | - |
| gene3614 | Chr | VFG013374(gi:16272852) | LOS | - |
| gene3617 | Chr | VFG015000(gi:15598845) | Alginate regulation | - |
| gene3631 | Chr | VFG014950(gi:15595963) | Alginate regulation | - |
| gene3638 | Chr | VFG016046(gi:15597609) | pyoverdine | - |
| gene3639 | Chr | VFG013069(gi:82778665) | Shu | - |
| gene3641 | Chr | VFG000925(gi:26246567) | Enterobactin | - |
| gene3642 | Chr | VFG013064(gi:82778670) | Shu | - |
| gene3657 | Chr | VFG005776(gi:22536834) | Beta-hemolysin/cytolysin | - |
| gene3677 | Chr | VFG000907(gi:26249408) | Hemolysin | - |
| gene3678 | Chr | VFG000934(gi:26246575) | Enterobactin | - |
| gene3696 | Chr | VFG010459(gi:52842133) | type IV pili | - |
| gene3708 | Chr | VFG013412(gi:16273070) | LOS | - |
| gene3771 | Chr | VFG000912(gi:26247116) | F1C fimbriae | - |
| gene3796 | Chr | VFG044172(gi:26250140) | Chu | - |
| gene3798 | Chr | VFG016041(gi:15597598) | pyoverdine | - |
| gene3801 | Chr | VFG044172(gi:26250140) | Chu | - |
| gene3802 | Chr | VFG044083(gi:15597454) | pyoverdine | - |
| gene3840 | Chr | VFG000842(gi:75994493) | Hemolysin | - |
| gene3858 | Chr | VFG015983(gi:15597589) | pyoverdine | - |
| gene3926 | Chr | VFG013418(gi:16273103) | LOS | - |
| gene3955 | Chr | VFG001864(gi:52841028) | Mip | - |
| gene3964 | Chr | VFG043209(gi:123442801) | peritrichous flagella | - |
| gene3987 | Chr | VFG044083(gi:15597454) | pyoverdine | - |
| gene4039 | Chr | VFG007138(gi:15600788) | VAS | - |
| gene4040 | Chr | VFG014733(gi:15598722) | Deoxyhexose linking sugar, 209 Da capping structure | - |
| gene4049 | Chr | VFG000917(gi:26250130) | Chu | - |
| gene4050 | Chr | VFG044083(gi:15597454) | pyoverdine | - |
| gene4069 | Chr | VFG044083(gi:15597454) | pyoverdine | - |
| gene4072 | Chr | VFG044083(gi:15597454) | pyoverdine | - |
| gene4089 | Chr | VFG045467(gi:53718898) | CdpA | - |
| gene4099 | Chr | VFG043209(gi:123442801) | peritrichous flagella | - |
| gene4149 | Chr | VFG000907(gi:26249408) | Hemolysin | - |
| gene4287 | Chr | VFG000033(gi:33591346) | LPS | - |
| gene4292 | Chr | VFG013508(gi:16273603) | LOS | - |
| gene4311 | Chr | VFG044083(gi:15597454) | pyoverdine | - |
| gene4319 | Chr | VFG000917(gi:26250130) | Chu | - |
| gene4338 | Chr | VFG002197(gi:29375537) | BopD | - |
| gene4359 | Chr | VFG015518(gi:15599410) | Phenazines biosynthesis | - |
| gene4360 | Chr | VFG005767(gi:22536831) | Beta-hemolysin/cytolysin | - |
| gene4369 | Chr | VFG009810(gi:15607898) | PhoP/R | - |
| gene4381 | Chr | VFG000332(gi:16273039) | LOS | - |
| gene4382 | Chr | VFG013400(gi:16273031) | LOS | - |
| gene4386 | Chr | VFG013286(gi:16272302) | LOS | - |
| gene4391 | Chr | VFG013496(gi:16273585) | LOS | - |
| gene4394 | Chr | VFG000330(gi:16272595) | LOS | - |
| gene4395 | Chr | VFG013315(gi:16272596) | LOS | - |
| gene4458 | Chr | VFG002197(gi:29375537) | BopD | - |
| gene4462 | Chr | VFG012509(gi:26247127) | Salmochelin | - |
| gene4464 | Chr | VFG005767(gi:22536831) | Beta-hemolysin/cytolysin | - |
| gene4470 | Chr | VFG009810(gi:15607898) | PhoP/R | - |
| p_gene0035 | p | VFG036992(gi:15677562) | MtrCDE | - |
| p_gene0036 | p | VFG036992(gi:15677562) | MtrCDE | - |
| p_gene0060 | p | VFG002302(gi:16766093) | Mig-14 | - |
| p_gene0062 | p | VFG002197(gi:29375537) | BopD | - |
| p_gene0070 | p | VFG000887(gi:26249421) | P fimbriae | - |
| p_gene0075 | p | VFG000885(gi:26249424) | P fimbriae | - |
| p_gene0076 | p | VFG000884(gi:26249425) | P fimbriae | - |
| p_gene0077 | p | VFG000884(gi:26249425) | P fimbriae | - |
| p_gene0078 | p | VFG000882(gi:26249427) | P fimbriae | - |
| p_gene0083 | p | VFG044081(gi:15597452) | pyoverdine | - |
| p_gene0108 | p | VFG000907(gi:26249408) | Hemolysin | - |
|  |  |  |  |  |
