# Supplementary material for: Seed-Borne Erwinia persicina Affects the Growth and Physiology of Alfalfa (Medicago sativa L.)
Source: Front Microbiol. 2022 May 26;13:891188. doi: 10.3389/fmicb.2022.891188 (PMC9178255; doi:10.3389/fmicb.2022.891188)
Supplement: Supplementary file 2 [file Table_2.DOCX]

| **TABLE S2** \| Identification of pathogenicity genes through interrogation of the *E. persicina* strain Cp2 genome with the PHI-base | | | | | |
| --- | --- | --- | --- | --- | --- |
| **Gene ID** | **Location** | **Hit Gene Name** | **PHI ID** | **Pathogen Species** | **Mutant Phenotype** |
| gene0006 | Chr | orf408 | PHI:612 | *Salmonella enterica* | reduced virulence |
| gene0013 | Chr | HspL | PHI:2657 | *Agrobacterium tumefaciens* | reduced virulence |
| gene0022 | Chr | OmpA1 | PHI:3937 | *Xanthomonas albilineans* | reduced virulence |
| gene0033 | Chr | fruR | PHI:6503 | *Streptococcus pyogenes* | unaffected pathogenicity |
| gene0034 | Chr | argD | PHI:3126 | *Erwinia amylovora* | reduced virulence |
| gene0035 | Chr | SOD2 | PHI:420 | *Cryptococcus gattii* | loss of pathogenicity |
| gene0036 | Chr | potA | PHI:6319 | *Streptococcus pneumoniae* | reduced virulence |
| gene0048 | Chr | Mpd1 | PHI:2249 | *Parastagonospora nodorum* | unaffected pathogenicity |
| gene0067 | Chr | fabG1 | PHI:5271 | *Ralstonia solanacearum* | lethal |
| gene0078 | Chr | potA | PHI:6319 | *Streptococcus pneumoniae* | reduced virulence |
| gene0086 | Chr | LptA | PHI:4910 | *Haemophilus ducreyi* | unaffected pathogenicity |
| gene0090 | Chr | potA | PHI:6319 | *Streptococcus pneumoniae* | reduced virulence |
| gene0091 | Chr | potA | PHI:6319 | *Streptococcus pneumoniae* | reduced virulence |
| gene0108 | Chr | MorA | PHI:4684 | *Pseudomonas aeruginosa* | increased virulence (hypervirulence) |
| gene0111 | Chr | ypo3991 | PHI:4189 | *Yersinia pestis* | reduced virulence |
| gene0119 | Chr | PMR1 | PHI:440 | *Candida albicans* | reduced virulence |
| gene0129 | Chr | PCK1 | PHI:424 | *Cryptococcus neoformans* | reduced virulence |
| gene0136 | Chr | ramA | PHI:5370 | *Salmonella enterica* | reduced virulence |
| gene0144 | Chr | kdpB | PHI:4712 | *Pseudomonas aeruginosa* | reduced virulence |
| gene0146 | Chr | kdpD | PHI:6313 | *Pseudomonas syringae* | reduced virulence |
| gene0150 | Chr | ramA | PHI:5370 | *Salmonella enterica* | reduced virulence |
| gene0154 | Chr | ybtQ | PHI:6913 | *Escherichia coli* | reduced virulence |
| gene0156 | Chr | fleQ | PHI:3219 | *Xanthomonas oryzae* | unaffected pathogenicity |
| gene0165 | Chr | pecS | PHI:4176 | *Dickeya solani* | increased virulence (hypervirulence) |
| gene0185 | Chr | ybtP | PHI:6912 | *Escherichia coli* | reduced virulence |
| gene0192 | Chr | Glr1 | PHI:4904 | *Candida albicans* | reduced virulence |
| gene0197 | Chr | vasH | PHI:3978 | *Aeromonas hydrophila* | effector (plant avirulence determinant) |
| gene0211 | Chr | Sdh1 | PHI:3914 | *Parastagonospora nodorum* | reduced virulence |
| gene0212 | Chr | AOX1 | PHI:199 | *Passalora fulva* | reduced virulence |
| gene0213 | Chr | DgcP | PHI:4663 | *Pseudomonas aeruginosa* | reduced virulence |
| gene0215 | Chr | MGG_04556 | PHI:881 | *Magnaporthe oryzae* | reduced virulence |
| gene0217 | Chr | MorA | PHI:4684 | *Pseudomonas aeruginosa* | increased virulence (hypervirulence) |
| gene0221 | Chr | pdhR | PHI:3135 | *Yersinia pseudotuberculosis* | reduced virulence |
| gene0225 | Chr | potA | PHI:6319 | *Streptococcus pneumoniae* | reduced virulence |
| gene0229 | Chr | MoSSADH | PHI:2145 | *Magnaporthe oryzae* | loss of pathogenicity |
| gene0242 | Chr | copA | PHI:5275 | *Vibrio tasmaniensis* | reduced virulence |
| gene0249 | Chr | MacB | PHI:3928 | *Salmonella enterica* | reduced virulence |
| gene0251 | Chr | rpoS | PHI:3336 | *Erwinia amylovora* | unaffected pathogenicity |
| gene0259 | Chr | potA | PHI:6319 | *Streptococcus pneumoniae* | reduced virulence |
| gene0260 | Chr | MacB | PHI:3928 | *Salmonella enterica* | reduced virulence |
| gene0264 | Chr | potA | PHI:6319 | *Streptococcus pneumoniae* | reduced virulence |
| gene0267 | Chr | GGT | PHI:3146 | *Helicobacter pylori* | unaffected pathogenicity |
| gene0269 | Chr | fabG1 | PHI:5271 | *Ralstonia solanacearum* | lethal |
| gene0275 | Chr | gntK | PHI:6701 | *Vibrio cholerae* | reduced virulence |
| gene0279 | Chr | treS | PHI:6732 | *Mycobacterium tuberculosis* | unaffected pathogenicity |
| gene0281 | Chr | glgC | PHI:6731 | *Mycobacterium tuberculosis* | reduced virulence |
| gene0284 | Chr | glpD | PHI:3272 | *Yersinia pestis* | unaffected pathogenicity |
| gene0287 | Chr | yihW | PHI:6545 | *Salmonella enterica* | unaffected pathogenicity |
| gene0290 | Chr | KSA1 | PHI:724 | *Fusarium graminearum* | unaffected pathogenicity |
| gene0296 | Chr | feoB | PHI:6941 | *Pseudomonas aeruginosa* | increased virulence (hypervirulence) |
| gene0298 | Chr | pnp | PHI:6080 | *Pseudomonas aeruginosa* | reduced virulence |
| gene0300 | Chr | ompR | PHI:2685 | *Salmonella enterica* | reduced virulence |
| gene0301 | Chr | envZ | PHI:2686 | *Salmonella enterica* | reduced virulence |
| gene0302 | Chr | PCK1 | PHI:424 | *Cryptococcus neoformans* | reduced virulence |
| gene0313 | Chr | PilQ | PHI:3827 | *Neisseria meningitidis* | reduced virulence |
| gene0315 | Chr | aroB | PHI:4250 | *Burkholderia glumae* | reduced virulence |
| gene0316 | Chr | damX | PHI:6729 | *Escherichia coli* | reduced virulence |
| gene0326 | Chr | argD | PHI:3126 | *Erwinia amylovora* | reduced virulence |
| gene0328 | Chr | Crp | PHI:4063 | *Yersinia pestis* | reduced virulence |
| gene0336 | Chr | potA | PHI:6319 | *Streptococcus pneumoniae* | reduced virulence |
| gene0342 | Chr | ybtQ | PHI:6913 | *Escherichia coli* | reduced virulence |
| gene0348 | Chr | FkpA | PHI:4583 | *Cronobacter turicensis* | reduced virulence |
| gene0353 | Chr | GzOB039 | PHI:1599 | *Fusarium graminearum* | unaffected pathogenicity |
| gene0361 | Chr | GzOB009 | PHI:1569 | *Fusarium graminearum* | lethal |
| gene0378 | Chr | secY | PHI:5257 | *Listeria monocytogenes* | increased virulence (hypervirulence) |
| gene0388 | Chr | rsmB | PHI:2694 | *Pectobacterium atrosepticum* | reduced virulence |
| gene0405 | Chr | pstB | PHI:3412 | *Xanthomonas citri* | loss of pathogenicity |
| gene0411 | Chr | PA3242 | PHI:3787 | *Pseudomonas aeruginosa* | increased virulence (hypervirulence) |
| gene0417 | Chr | GzC2H048 | PHI:1385 | *Fusarium graminearum* | unaffected pathogenicity |
| gene0428 | Chr | RNase E | PHI:3723 | *Salmonella enterica* | reduced virulence |
| gene0433 | Chr | oqxA | PHI:6448 | *Klebsiella pneumoniae* | reduced virulence |
| gene0436 | Chr | Sdh1 | PHI:3914 | *Parastagonospora nodorum* | reduced virulence |
| gene0442 | Chr | mdh | PHI:2959 | *Edwardsiella ictaluri* | reduced virulence |
| gene0443 | Chr | MoRga7 | PHI:3067 | *Magnaporthe oryzae* | unaffected pathogenicity |
| gene0444 | Chr | HtrA | PHI:6358 | *Haemophilus parasuis* | reduced virulence |
| gene0454 | Chr | rpfC | PHI:3592 | *Xanthomonas hortorum* | reduced virulence |
| gene0458 | Chr | ptsN | PHI:3133 | *Yersinia pseudotuberculosis* | reduced virulence |
| gene0459 | Chr | yhbH | PHI:3674 | *Erwinia amylovora* | loss of pathogenicity |
| gene0460 | Chr | rpoN | PHI:2906 | *Erwinia amylovora* | loss of pathogenicity |
| gene0461 | Chr | StpC | PHI:6324 | *Staphylococcus aureus* | unaffected pathogenicity |
| gene0467 | Chr | ABC3 | PHI:1018 | *Magnaporthe oryzae* | loss of pathogenicity |
| gene0481 | Chr | ybdB | PHI:6270 | *Escherichia coli* | lethal |
| gene0483 | Chr | entB | PHI:6565 | *Klebsiella pneumoniae* | unaffected pathogenicity |
| gene0484 | Chr | pchD | PHI:6938 | *Pseudomonas aeruginosa* | loss of pathogenicity |
| gene0486 | Chr | qseC | PHI:3709 | *Pectobacterium carotovorum* | reduced virulence |
| gene0487 | Chr | PmrA | PHI:4497 | *Salmonella enterica* | increased virulence (hypervirulence) |
| gene0492 | Chr | GzOB006 | PHI:1566 | *Fusarium graminearum* | lethal |
| gene0499 | Chr | PspK | PHI:6281 | *Streptococcus pneumoniae* | reduced virulence |
| gene0503 | Chr | pnp | PHI:3878 | *Salmonella enterica* | reduced virulence |
| gene0505 | Chr | CshA | PHI:6510 | *Listeria monocytogenes* | unaffected pathogenicity |
| gene0520 | Chr | GzMyb016 | PHI:1552 | *Fusarium graminearum* | unaffected pathogenicity |
| gene0521 | Chr | treS | PHI:6732 | *Mycobacterium tuberculosis* | unaffected pathogenicity |
| gene0522 | Chr | HEX1 | PHI:4987 | *Candida albicans* | reduced virulence |
| gene0527 | Chr | argF | PHI:2634 | *Staphylococcus aureus* | unaffected pathogenicity |
| gene0540 | Chr | fabG1 | PHI:5271 | *Ralstonia solanacearum* | lethal |
| gene0572 | Chr | fabG1 | PHI:5271 | *Ralstonia solanacearum* | lethal |
| gene0577 | Chr | oqxA | PHI:6448 | *Klebsiella pneumoniae* | reduced virulence |
| gene0578 | Chr | oqxB | PHI:6449 | *Klebsiella pneumoniae* | reduced virulence |
| gene0581 | Chr | copA | PHI:5275 | *Vibrio tasmaniensis* | reduced virulence |
| gene0588 | Chr | narL | PHI:3620 | *Mycobacterium tuberculosis* | unaffected pathogenicity |
| gene0594 | Chr | potA | PHI:6319 | *Streptococcus pneumoniae* | reduced virulence |
| gene0602 | Chr | pbpX | PHI:4692 | *Listeria monocytogenes* | reduced virulence |
| gene0607 | Chr | aphB | PHI:5548 | *Vibrio cholerae* | reduced virulence |
| gene0608 | Chr | aer | PHI:6979 | *Salmonella enterica* | reduced virulence |
| gene0609 | Chr | IPMDH | PHI:415 | *Parastagonospora nodorum* | loss of pathogenicity |
| gene0611 | Chr | rpoS | PHI:3336 | *Erwinia amylovora* | unaffected pathogenicity |
| gene0622 | Chr | rfaE | PHI:3147 | *Haemophilus parasuis* | reduced virulence |
| gene0623 | Chr | yqiC | PHI:6697 | *Salmonella enterica* | reduced virulence |
| gene0629 | Chr | AbuO | PHI:4996 | *Acinetobacter baumannii* | reduced virulence |
| gene0639 | Chr | FbaA | PHI:6903 | *Staphylococcus aureus* | reduced virulence |
| gene0640 | Chr | FbaA | PHI:6903 | *Staphylococcus aureus* | reduced virulence |
| gene0645 | Chr | GyrA | PHI:824 | *Burkholderia glumae* | chemistry target: resistance to chemical |
| gene0646 | Chr | mrLPAAT1 | PHI:6380 | *Metarhizium robertsii* | reduced virulence |
| gene0652 | Chr | argD | PHI:3126 | *Erwinia amylovora* | reduced virulence |
| gene0659 | Chr | gigX4 | PHI:6956 | *Xanthomonas oryzae* | increased virulence (hypervirulence) |
| gene0665 | Chr | MorA | PHI:4684 | *Pseudomonas aeruginosa* | increased virulence (hypervirulence) |
| gene0666 | Chr | Vatr2 | PHI:3028 | *Clavibacter michiganensis* | reduced virulence |
| gene0685 | Chr | PA2206 | PHI:2669 | *Pseudomonas aeruginosa* | reduced virulence |
| gene0686 | Chr | Ss-odc2 | PHI:4509 | *Sclerotinia sclerotiorum* | reduced virulence |
| gene0687 | Chr | potA | PHI:6319 | *Streptococcus pneumoniae* | reduced virulence |
| gene0694 | Chr | ccpE | PHI:5242 | *Staphylococcus aureus* | increased virulence (hypervirulence) |
| gene0713 | Chr | chbC | PHI:6529 | *Borrelia burgdorferi* | unaffected pathogenicity |
| gene0716 | Chr | WzzfepE | PHI:3727 | *Salmonella enterica* | loss of pathogenicity |
| gene0720 | Chr | trg | PHI:6982 | *Salmonella enterica* | reduced virulence |
| gene0722 | Chr | MorA | PHI:4684 | *Pseudomonas aeruginosa* | increased virulence (hypervirulence) |
| gene0723 | Chr | lrp | PHI:6497 | *Xenorhabdus nematophila* | reduced virulence |
| gene0726 | Chr | pstB | PHI:3412 | *Xanthomonas citri* | loss of pathogenicity |
| gene0727 | Chr | niuD | PHI:6849 | *Helicobacter pylori* | reduced virulence |
| gene0728 | Chr | niuD | PHI:6849 | *Helicobacter pylori* | reduced virulence |
| gene0730 | Chr | fiuA | PHI:6373 | *Pseudomonas aeruginosa* | reduced virulence |
| gene0733 | Chr | ecf6 | PHI:2876 | *Pseudomonas syringae* | unaffected pathogenicity |
| gene0735 | Chr | FVEG_12531 | PHI:3383 | *Fusarium verticillioides* | unaffected pathogenicity |
| gene0736 | Chr | pdhR | PHI:3135 | *Yersinia pseudotuberculosis* | reduced virulence |
| gene0738 | Chr | ireA | PHI:6654 | *Escherichia coli* | unaffected pathogenicity |
| gene0742 | Chr | potA | PHI:6319 | *Streptococcus pneumoniae* | reduced virulence |
| gene0744 | Chr | MoDeam | PHI:5472 | *Magnaporthe oryzae* | reduced virulence |
| gene0763 | Chr | MorA | PHI:4684 | *Pseudomonas aeruginosa* | increased virulence (hypervirulence) |
| gene0769 | Chr | tcp | PHI:6980 | *Salmonella enterica* | reduced virulence |
| gene0789 | Chr | pilT | PHI:6363 | *Pantoea ananatis* | reduced virulence |
| gene0796 | Chr | iolT | PHI:6381 | *Legionella pneumophila* | reduced virulence |
| gene0797 | Chr | MGG_00383 | PHI:877 | *Magnaporthe oryzae* | reduced virulence |
| gene0806 | Chr | GAPDH | PHI:6904 | *Staphylococcus aureus* | reduced virulence |
| gene0808 | Chr | FbaA | PHI:6903 | *Staphylococcus aureus* | reduced virulence |
| gene0820 | Chr | ubiI | PHI:7028 | *Escherichia coli* | reduced virulence |
| gene0821 | Chr | ubiI | PHI:7028 | *Escherichia coli* | reduced virulence |
| gene0825 | Chr | gcvP | PHI:2961 | *Edwardsiella ictaluri* | reduced virulence |
| gene0826 | Chr | fabG1 | PHI:5271 | *Ralstonia solanacearum* | lethal |
| gene0832 | Chr | ompR | PHI:2685 | *Salmonella enterica* | reduced virulence |
| gene0833 | Chr | kdpD | PHI:6313 | *Pseudomonas syringae* | reduced virulence |
| gene0840 | Chr | MntE | PHI:4652 | *Streptococcus pyogenes* | unaffected pathogenicity |
| gene0841 | Chr | GzOB034 | PHI:1594 | *Fusarium graminearum* | unaffected pathogenicity |
| gene0845 | Chr | PA2206 | PHI:2669 | *Pseudomonas aeruginosa* | reduced virulence |
| gene0848 | Chr | chbC | PHI:6529 | *Borrelia burgdorferi* | unaffected pathogenicity |
| gene0857 | Chr | adeI | PHI:6386 | *Acinetobacter baumannii* | unaffected pathogenicity |
| gene0858 | Chr | acrB | PHI:6451 | *Klebsiella pneumoniae* | reduced virulence |
| gene0859 | Chr | adeK | PHI:6388 | *Acinetobacter baumannii* | unaffected pathogenicity |
| gene0860 | Chr | adeA | PHI:6263 | *Acinetobacter baumannii* | unaffected pathogenicity |
| gene0861 | Chr | oqxB | PHI:6449 | *Klebsiella pneumoniae* | reduced virulence |
| gene0862 | Chr | oqxB | PHI:6449 | *Klebsiella pneumoniae* | reduced virulence |
| gene0863 | Chr | adeK | PHI:6388 | *Acinetobacter baumannii* | unaffected pathogenicity |
| gene0864 | Chr | AKT1 | PHI:133 | *Alternaria alternata* | loss of pathogenicity |
| gene0905 | Chr | barA | PHI:562 | *Salmonella enterica* | unaffected pathogenicity |
| gene0906 | Chr | relA | PHI:4166 | *Vibrio cholerae* | unaffected pathogenicity |
| gene0912 | Chr | tcp | PHI:6980 | *Salmonella enterica* | reduced virulence |
| gene0917 | Chr | pstB | PHI:3412 | *Xanthomonas citri* | loss of pathogenicity |
| gene0928 | Chr | raxP (cysD) | PHI:1138 | *Xanthomonas oryzae* | increased virulence (hypervirulence) |
| gene0930 | Chr | MET3 | PHI:265 | *Cryptococcus neoformans* | loss of pathogenicity |
| gene0945 | Chr | pimt | PHI:6187 | *Salmonella enterica* | reduced virulence |
| gene0947 | Chr | rpoS | PHI:3336 | *Erwinia amylovora* | unaffected pathogenicity |
| gene0948 | Chr | citB | PHI:5066 | *Xanthomonas oryzae* | reduced virulence |
| gene0955 | Chr | LssB | PHI:4975 | *Legionella pneumophila* | reduced virulence |
| gene0966 | Chr | recA | PHI:4702 | *Streptococcus pneumoniae* | reduced virulence |
| gene0969 | Chr | csrA | PHI:2682 | *Salmonella enterica* | reduced virulence |
| gene0978 | Chr | LuxS | PHI:3731 | *Escherichia coli* | reduced virulence |
| gene0999 | Chr | yfiA | PHI:3675 | *Erwinia amylovora* | unaffected pathogenicity |
| gene1003 | Chr | T6SS2 | PHI:4559 | *Escherichia coli* | effector (plant avirulence determinant) |
| gene1011 | Chr | Pat | PHI:5571 | *Salmonella enterica* | reduced virulence |
| gene1017 | Chr | MprA | PHI:5407 | *Escherichia coli* | loss of pathogenicity |
| gene1022 | Chr | pstB | PHI:3412 | *Xanthomonas citri* | loss of pathogenicity |
| gene1053 | Chr | smpB | PHI:4218 | *Escherichia coli* | reduced virulence |
| gene1060 | Chr | tsr | PHI:6981 | *Salmonella enterica* | reduced virulence |
| gene1065 | Chr | CshA | PHI:6510 | *Listeria monocytogenes* | unaffected pathogenicity |
| gene1067 | Chr | frdA | PHI:2960 | *Edwardsiella ictaluri* | reduced virulence |
| gene1068 | Chr | rpoE | PHI:2680 | *Salmonella enterica* | reduced virulence |
| gene1074 | Chr | RNase III | PHI:3724 | *Salmonella enterica* | reduced virulence |
| gene1083 | Chr | orf408 | PHI:612 | *Salmonella enterica* | reduced virulence |
| gene1091 | Chr | FOS1 | PHI:253 | *Aspergillus fumigatus* | reduced virulence |
| gene1093 | Chr | luxO | PHI:4100 | *Vibrio harveyi* | reduced virulence |
| gene1097 | Chr | BCFHG1 | PHI:2304 | *Botrytis cinerea* | unaffected pathogenicity |
| gene1098 | Chr | glyA | PHI:2962 | *Edwardsiella ictaluri* | reduced virulence |
| gene1105 | Chr | IscR | PHI:3043 | *Yersinia pseudotuberculosis* | reduced virulence |
| gene1106 | Chr | nifS | PHI:4210 | *Mycoplasma agalactiae* | loss of pathogenicity |
| gene1109 | Chr | sseA | PHI:609 | *Salmonella enterica* | reduced virulence |
| gene1112 | Chr | XC_2203 | PHI:3945 | *Xanthomonas campestris* | reduced virulence |
| gene1125 | Chr | IMPDH | PHI:4071 | *Streptococcus suis* | reduced virulence |
| gene1126 | Chr | gua1 | PHI:6871 | *Cryptococcus neoformans* | loss of pathogenicity |
| gene1143 | Chr | DipA | PHI:3042 | *Pseudomonas aeruginosa* | reduced virulence |
| gene1146 | Chr | ppk | PHI:5063 | *Proteus mirabilis* | reduced virulence |
| gene1148 | Chr | Rv0930 | PHI:3635 | *Mycobacterium tuberculosis* | unaffected pathogenicity |
| gene1149 | Chr | pstB | PHI:3412 | *Xanthomonas citri* | loss of pathogenicity |
| gene1152 | Chr | iaaH-1 | PHI:4171 | *Pseudomonas savastanoi* | reduced virulence |
| gene1156 | Chr | ADE5 | PHI:744 | *Fusarium graminearum* | reduced virulence |
| gene1163 | Chr | cgsB | PHI:3647 | *Brucella melitensis* | increased virulence (hypervirulence) |
| gene1177 | Chr | AcrD | PHI:4043 | *Erwinia amylovora* | unaffected pathogenicity |
| gene1178 | Chr | degU | PHI:4690 | *Listeria monocytogenes* | reduced virulence |
| gene1183 | Chr | orf408 | PHI:612 | *Salmonella enterica* | reduced virulence |
| gene1188 | Chr | StpC | PHI:6324 | *Staphylococcus aureus* | unaffected pathogenicity |
| gene1194 | Chr | SrrAB | PHI:5466 | *Staphylococcus aureus* | reduced virulence |
| gene1195 | Chr | tcrY | PHI:3616 | *Mycobacterium tuberculosis* | increased virulence (hypervirulence) |
| gene1216 | Chr | potC | PHI:6321 | *Streptococcus pneumoniae* | reduced virulence |
| gene1218 | Chr | potA | PHI:6319 | *Streptococcus pneumoniae* | reduced virulence |
| gene1220 | Chr | CpxR | PHI:4523 | *Escherichia coli* | reduced virulence |
| gene1221 | Chr | trcS | PHI:3617 | *Mycobacterium tuberculosis* | increased virulence (hypervirulence) |
| gene1223 | Chr | glcA | PHI:6308 | *Staphylococcus aureus* | reduced virulence |
| gene1234 | Chr | OxyR | PHI:3714 | *Escherichia coli* | reduced virulence |
| gene1236 | Chr | THR1 | PHI:59 | *Colletotrichum lagenarium* | reduced virulence |
| gene1254 | Chr | 1910HK (ssu05 1911) | PHI:6896 | *Streptococcus suis* | reduced virulence |
| gene1256 | Chr | iaaH-1 | PHI:4171 | *Pseudomonas savastanoi* | reduced virulence |
| gene1283 | Chr | MAK1 | PHI:112 | *Nectria haematococca* | reduced virulence |
| gene1291 | Chr | oqxA | PHI:6448 | *Klebsiella pneumoniae* | reduced virulence |
| gene1302 | Chr | tsr | PHI:6981 | *Salmonella enterica* | reduced virulence |
| gene1310 | Chr | T6SS1 | PHI:4558 | *Escherichia coli* | effector (plant avirulence determinant) |
| gene1314 | Chr | tssM | PHI:5090 | *Acinetobacter baumannii* | effector (plant avirulence determinant) |
| gene1321 | Chr | StpC | PHI:6324 | *Staphylococcus aureus* | unaffected pathogenicity |
| gene1330 | Chr | vacJ | PHI:6900 | *Haemophilus parasuis* | reduced virulence |
| gene1333 | Chr | MoAcat1 | PHI:5082 | *Magnaporthe oryzae* | reduced virulence |
| gene1344 | Chr | MorA | PHI:4684 | *Pseudomonas aeruginosa* | increased virulence (hypervirulence) |
| gene1354 | Chr | KSA1 | PHI:724 | *Fusarium graminearum* | unaffected pathogenicity |
| gene1362 | Chr | ABC4 | PHI:1017 | *Magnaporthe oryzae* | reduced virulence |
| gene1376 | Chr | PidR | PHI:2440 | *Burkholderia glumae* | reduced virulence |
| gene1377 | Chr | QseC | PHI:6883 | *Salmonella enterica* | reduced virulence |
| gene1386 | Chr | ADE4 | PHI:502 | *Saccharomyces cerevisiae* | reduced virulence |
| gene1391 | Chr | pstB | PHI:3412 | *Xanthomonas citri* | loss of pathogenicity |
| gene1395 | Chr | argD | PHI:3126 | *Erwinia amylovora* | reduced virulence |
| gene1396 | Chr | eutD | PHI:6516 | *Mycoplasma agalactiae* | reduced virulence |
| gene1404 | Chr | RovM | PHI:5089 | *Yersinia pestis* | reduced virulence |
| gene1407 | Chr | NOS1 | PHI:445 | *Fusarium graminearum* | reduced virulence |
| gene1419 | Chr | opgG | PHI:5564 | *Dickeya dadantii* | reduced virulence |
| gene1432 | Chr | GyrA | PHI:824 | *Burkholderia glumae* | chemistry target |
| gene1435 | Chr | rcsC11 | PHI:3011 | *Salmonella enterica* | unaffected pathogenicity |
| gene1436 | Chr | rcsB | PHI:2499 | *Erwinia amylovora* | loss of pathogenicity |
| gene1437 | Chr | rcsD | PHI:2501 | *Erwinia amylovora* | loss of pathogenicity |
| gene1445 | Chr | LssB | PHI:4975 | *Legionella pneumophila* | reduced virulence |
| gene1447 | Chr | ompK36 | PHI:2872 | *Klebsiella pneumoniae* | reduced virulence |
| gene1451 | Chr | potA | PHI:6319 | *Streptococcus pneumoniae* | reduced virulence |
| gene1452 | Chr | pstB | PHI:3412 | *Xanthomonas citri* | loss of pathogenicity |
| gene1474 | Chr | potA | PHI:6319 | *Streptococcus pneumoniae* | reduced virulence |
| gene1478 | Chr | rpfR | PHI:5504 | *Cronobacter turicensis* | unaffected pathogenicity |
| gene1487 | Chr | fruK | PHI:6527 | *Borrelia burgdorferi* | unaffected pathogenicity |
| gene1489 | Chr | ANP1 | PHI:3793 | *Candida glabrata* | increased virulence (hypervirulence) |
| gene1493 | Chr | niuD | PHI:6849 | *Helicobacter pylori* | reduced virulence |
| gene1494 | Chr | potA | PHI:6319 | *Streptococcus pneumoniae* | reduced virulence |
| gene1496 | Chr | gnoA | PHI:3277 | *Aspergillus fumigatus* | unaffected pathogenicity |
| gene1501 | Chr | CcpA | PHI:4564 | *Enterococcus faecium* | reduced virulence |
| gene1503 | Chr | StpC | PHI:6324 | *Staphylococcus aureus* | unaffected pathogenicity |
| gene1519 | Chr | aphB | PHI:5548 | *Vibrio cholerae* | reduced virulence |
| gene1535 | Chr | hp0169 | PHI:6964 | *Helicobacter pylori* | reduced virulence |
| gene1536 | Chr | SrrAB | PHI:5466 | *Staphylococcus aureus* | reduced virulence |
| gene1537 | Chr | tcrY | PHI:3616 | *Mycobacterium tuberculosis* | increased virulence (hypervirulence) |
| gene1539 | Chr | oqxB | PHI:6449 | *Klebsiella pneumoniae* | reduced virulence |
| gene1540 | Chr | oqxB | PHI:6449 | *Klebsiella pneumoniae* | reduced virulence |
| gene1541 | Chr | oqxA | PHI:6448 | *Klebsiella pneumoniae* | reduced virulence |
| gene1546 | Chr | MorA | PHI:4684 | *Pseudomonas aeruginosa* | increased virulence (hypervirulence) |
| gene1549 | Chr | magA | PHI:2652 | *Klebsiella pneumoniae* | reduced virulence |
| gene1552 | Chr | cps2E | PHI:6138 | *Streptococcus suis* | reduced virulence |
| gene1555 | Chr | Ams | PHI:2471 | *Erwinia amylovora* | loss of pathogenicity |
| gene1558 | Chr | cps2J | PHI:6140 | *Streptococcus suis* | reduced virulence |
| gene1565 | Chr | galU | PHI:3112 | *Xanthomonas campestris* | loss of pathogenicity |
| gene1566 | Chr | Lmepi | PHI:1133 | *Leptosphaeria maculans* | loss of pathogenicity |
| gene1571 | Chr | gigX1 | PHI:6953 | *Xanthomonas oryzae* | increased virulence (hypervirulence) |
| gene1580 | Chr | WzzST | PHI:3728 | *Salmonella enterica* | loss of pathogenicity |
| gene1585 | Chr | hisB | PHI:2516 | *Aspergillus fumigatus* | lethal |
| gene1588 | Chr | prt | PHI:5109 | *Salmonella enterica* | reduced virulence |
| gene1608 | Chr | glcA | PHI:6308 | *Staphylococcus aureus* | reduced virulence |
| gene1610 | Chr | PafR | PHI:4630 | *Escherichia coli* | reduced virulence |
| gene1614 | Chr | sufC | PHI:6894 | *Salmonella enterica* | unaffected pathogenicity |
| gene1620 | Chr | potA | PHI:6319 | *Streptococcus pneumoniae* | reduced virulence |
| gene1625 | Chr | katG | PHI:6089 | *Acinetobacter nosocomialis* | increased virulence (hypervirulence) |
| gene1637 | Chr | niuD | PHI:6849 | *Helicobacter pylori* | reduced virulence |
| gene1639 | Chr | pstB | PHI:3412 | *Xanthomonas citri* | loss of pathogenicity |
| gene1640 | Chr | pvdL | PHI:6991 | *Pseudomonas aeruginosa* | loss of pathogenicity |
| gene1642 | Chr | Fes | PHI:4859 | *Escherichia coli* | unaffected pathogenicity |
| gene1643 | Chr | fiuA | PHI:6373 | *Pseudomonas aeruginosa* | reduced virulence |
| gene1645 | Chr | fiuA | PHI:6373 | *Pseudomonas aeruginosa* | reduced virulence |
| gene1647 | Chr | fleQ | PHI:3219 | *Xanthomonas oryzae* | unaffected pathogenicity |
| gene1664 | Chr | pyk | PHI:6684 | *Brucella abortus* | reduced virulence |
| gene1676 | Chr | potA | PHI:6319 | *Streptococcus pneumoniae* | reduced virulence |
| gene1684 | Chr | pchD | PHI:6938 | *Pseudomonas aeruginosa* | loss of pathogenicity |
| gene1689 | Chr | pamA | PHI:3090 | *Paenibacillus larvae* | reduced virulence |
| gene1691 | Chr | fiuA | PHI:6373 | *Pseudomonas aeruginosa* | reduced virulence |
| gene1694 | Chr | VdSge1 | PHI:2731 | *Verticillium dahliae* | loss of pathogenicity |
| gene1695 | Chr | F-avi4330 | PHI:2895 | *Agrobacterium vitis* | loss of pathogenicity |
| gene1696 | Chr | ccpE | PHI:5242 | *Staphylococcus aureus* | increased virulence (hypervirulence) |
| gene1701 | Chr | OxyR | PHI:3714 | *Escherichia coli* | reduced virulence |
| gene1708 | Chr | PmrA | PHI:3599 | *Coxiella burnetii* | reduced virulence |
| gene1709 | Chr | QseC | PHI:6883 | *Salmonella enterica* | reduced virulence |
| gene1718 | Chr | StpC | PHI:6324 | *Staphylococcus aureus* | unaffected pathogenicity |
| gene1720 | Chr | SoxR | PHI:3092 | *Pantoea stewartii* | reduced virulence |
| gene1721 | Chr | ccpE | PHI:5242 | *Staphylococcus aureus* | increased virulence (hypervirulence) |
| gene1723 | Chr | Sdh1 | PHI:3914 | *Parastagonospora nodorum* | reduced virulence |
| gene1725 | Chr | MoSSADH | PHI:2145 | *Magnaporthe oryzae* | loss of pathogenicity |
| gene1731 | Chr | ybtQ | PHI:6913 | *Escherichia coli* | reduced virulence |
| gene1736 | Chr | argD | PHI:3126 | *Erwinia amylovora* | reduced virulence |
| gene1737 | Chr | lrp | PHI:6497 | *Xenorhabdus nematophila* | reduced virulence |
| gene1745 | Chr | expR | PHI:4178 | *Dickeya solani* | reduced virulence |
| gene1751 | Chr | ABC2 | PHI:391 | *Magnaporthe oryzae* | unaffected pathogenicity |
| gene1752 | Chr | MacB | PHI:3928 | *Salmonella enterica* | reduced virulence |
| gene1760 | Chr | potA | PHI:6319 | *Streptococcus pneumoniae* | reduced virulence |
| gene1773 | Chr | MacB | PHI:3928 | *Salmonella enterica* | reduced virulence |
| gene1783 | Chr | pobA | PHI:5404 | *Xanthomonas campestris* | reduced virulence |
| gene1787 | Chr | pstB | PHI:3412 | *Xanthomonas citri* | loss of pathogenicity |
| gene1794 | Chr | tcp | PHI:6980 | *Salmonella enterica* | reduced virulence |
| gene1798 | Chr | chbC | PHI:6529 | *Borrelia burgdorferi* | unaffected pathogenicity |
| gene1803 | Chr | potA | PHI:6319 | *Streptococcus pneumoniae* | reduced virulence |
| gene1809 | Chr | glcA | PHI:6308 | *Staphylococcus aureus* | reduced virulence |
| gene1813 | Chr | tsr | PHI:6981 | *Salmonella enterica* | reduced virulence |
| gene1814 | Chr | DgcP | PHI:4663 | *Pseudomonas aeruginosa* | reduced virulence |
| gene1828 | Chr | aphB | PHI:5548 | *Vibrio cholerae* | reduced virulence |
| gene1829 | Chr | MoHYR1 | PHI:2153 | *Magnaporthe oryzae* | reduced virulence |
| gene1834 | Chr | fabG1 | PHI:5271 | *Ralstonia solanacearum* | lethal |
| gene1852 | Chr | ybtQ | PHI:6913 | *Escherichia coli* | reduced virulence |
| gene1877 | Chr | fliD | PHI:6733 | *Escherichia coli* | unaffected pathogenicity |
| gene1878 | Chr | fliC | PHI:4614 | *Edwardsiella tarda* | increased virulence (hypervirulence) |
| gene1879 | Chr | gigX10 | PHI:6962 | *Xanthomonas oryzae* | increased virulence (hypervirulence) |
| gene1881 | Chr | gigX1 | PHI:6953 | *Xanthomonas oryzae* | increased virulence (hypervirulence) |
| gene1888 | Chr | rpoS | PHI:2683 | *Salmonella enterica* | reduced virulence |
| gene1889 | Chr | FliZ | PHI:3922 | *Xenorhabdus nematophila* | reduced virulence |
| gene1897 | Chr | Pot13p | PHI:3689 | *Candida albicans* | unaffected pathogenicity |
| gene1900 | Chr | RovM | PHI:5089 | *Yersinia pestis* | reduced virulence |
| gene1921 | Chr | MoSSADH | PHI:2145 | *Magnaporthe oryzae* | loss of pathogenicity |
| gene1923 | Chr | cps2J | PHI:6140 | *Streptococcus suis* | reduced virulence |
| gene1932 | Chr | glcA | PHI:6308 | *Staphylococcus aureus* | reduced virulence |
| gene1934 | Chr | VC1295 | PHI:3232 | *Vibrio cholerae* | unaffected pathogenicity |
| gene1936 | Chr | otsA | PHI:4144 | *Xanthomonas citri* | reduced virulence |
| gene1938 | Chr | ?hD | PHI:4075 | *Serratia marcescens* | unaffected pathogenicity |
| gene1940 | Chr | motA | PHI:6361 | *Pantoea ananatis* | reduced virulence |
| gene1941 | Chr | motB | PHI:6534 | *Escherichia coli* | reduced virulence |
| gene1942 | Chr | cheA | PHI:6535 | *Escherichia coli* | unaffected pathogenicity |
| gene1943 | Chr | cheW | PHI:6536 | *Escherichia coli* | unaffected pathogenicity |
| gene1944 | Chr | tsr | PHI:6981 | *Salmonella enterica* | reduced virulence |
| gene1945 | Chr | tsr | PHI:6981 | *Salmonella enterica* | reduced virulence |
| gene1946 | Chr | tsr | PHI:6981 | *Salmonella enterica* | reduced virulence |
| gene1947 | Chr | tsr | PHI:6981 | *Salmonella enterica* | reduced virulence |
| gene1949 | Chr | cheB | PHI:6461 | *Salmonella enterica* | reduced virulence |
| gene1950 | Chr | FOS1 | PHI:253 | *Aspergillus fumigatus* | reduced virulence |
| gene1952 | Chr | sctU | PHI:4470 | *Burkholderia pseudomallei* | reduced virulence |
| gene1953 | Chr | ascV | PHI:6829 | *Aeromonas salmonicida* | reduced virulence |
| gene1974 | Chr | rarA | PHI:6447 | *Klebsiella pneumoniae* | reduced virulence |
| gene1975 | Chr | znuB | PHI:5013 | *Yersinia pseudotuberculosis* | reduced virulence |
| gene1976 | Chr | potA | PHI:6319 | *Streptococcus pneumoniae* | reduced virulence |
| gene1978 | Chr | ypo2062 | PHI:4185 | *Yersinia pestis* | reduced virulence |
| gene1981 | Chr | pyk | PHI:6684 | *Brucella abortus* | reduced virulence |
| gene1986 | Chr | eda | PHI:6699 | *Vibrio cholerae* | reduced virulence |
| gene1991 | Chr | Oligopeptidase B | PHI:2580 | *Trypanosoma cruzi* | reduced virulence |
| gene2002 | Chr | gpT | PHI:3319 | *Haemophilus parasuis* | reduced virulence |
| gene2046 | Chr | prc | PHI:5547 | *Xanthomonas campestris* | reduced virulence |
| gene2049 | Chr | kdgR | PHI:4175 | *Dickeya solani* | unaffected pathogenicity |
| gene2053 | Chr | CspV | PHI:6372 | *Vibrio cholerae* | unaffected pathogenicity |
| gene2060 | Chr | DipA | PHI:3042 | *Pseudomonas aeruginosa* | reduced virulence |
| gene2067 | Chr | yeaZ | PHI:6870 | *Vibrio harveyi* | reduced virulence |
| gene2069 | Chr | pvdL | PHI:6991 | *Pseudomonas aeruginosa* | loss of pathogenicity |
| gene2079 | Chr | gntR | PHI:6702 | *Vibrio cholerae* | reduced virulence |
| gene2084 | Chr | gumD | PHI:2937 | *Xylella fastidiosa* | loss of pathogenicity |
| gene2093 | Chr | GAPDH | PHI:6904 | *Staphylococcus aureus* | reduced virulence |
| gene2111 | Chr | SKN7 | PHI:450 | *Cryptococcus neoformans* | reduced virulence |
| gene2112 | Chr | galU | PHI:3112 | *Xanthomonas campestris* | loss of pathogenicity |
| gene2113 | Chr | UGD1 | PHI:387 | *Cryptococcus neoformans* | loss of pathogenicity |
| gene2115 | Chr | hns | PHI:6097 | *Klebsiella pneumoniae* | increased virulence (hypervirulence) |
| gene2124 | Chr | potA | PHI:6319 | *Streptococcus pneumoniae* | reduced virulence |
| gene2125 | Chr | potA | PHI:6319 | *Streptococcus pneumoniae* | reduced virulence |
| gene2131 | Chr | tonB | PHI:6651 | *Klebsiella pneumoniae* | unaffected pathogenicity |
| gene2138 | Chr | TRPS | PHI:2517 | *Aspergillus fumigatus* | lethal |
| gene2139 | Chr | TRPS | PHI:2517 | *Aspergillus fumigatus* | lethal |
| gene2151 | Chr | fabG1 | PHI:5271 | *Ralstonia solanacearum* | lethal |
| gene2159 | Chr | SoxR | PHI:3092 | *Pantoea stewartii* | reduced virulence |
| gene2172 | Chr | rpfF | PHI:5503 | *Cronobacter turicensis* | reduced virulence |
| gene2173 | Chr | rpfR | PHI:5504 | *Cronobacter turicensis* | unaffected pathogenicity |
| gene2178 | Chr | pafP | PHI:4631 | *Escherichia coli* | reduced virulence |
| gene2181 | Chr | SoxR | PHI:3092 | *Pantoea stewartii* | reduced virulence |
| gene2184 | Chr | potA | PHI:6319 | *Streptococcus pneumoniae* | reduced virulence |
| gene2185 | Chr | ybtP | PHI:6912 | *Escherichia coli* | reduced virulence |
| gene2190 | Chr | LuxO | PHI:708 | *Vibrio cholerae* | reduced virulence |
| gene2197 | Chr | TyrR | PHI:4563 | *Yersinia pestis* | reduced virulence |
| gene2203 | Chr | PafR | PHI:4630 | *Escherichia coli* | reduced virulence |
| gene2204 | Chr | ABC4 | PHI:1017 | *Magnaporthe oryzae* | reduced virulence |
| gene2220 | Chr | ssuA | PHI:4028 | *Xanthomonas citri* | reduced virulence |
| gene2221 | Chr | MoSSADH | PHI:2145 | *Magnaporthe oryzae* | loss of pathogenicity |
| gene2236 | Chr | ramA | PHI:5370 | *Salmonella enterica* | reduced virulence |
| gene2240 | Chr | potA | PHI:6319 | *Streptococcus pneumoniae* | reduced virulence |
| gene2257 | Chr | PXO_00987 | PHI:4956 | *Xanthomonas oryzae* | reduced virulence |
| gene2259 | Chr | Mqo | PHI:6190 | *Staphylococcus aureus* | reduced virulence |
| gene2265 | Chr | vasH | PHI:3978 | *Aeromonas hydrophila* | effector (plant avirulence determinant) |
| gene2267 | Chr | adhT | PHI:6517 | *Mycoplasma agalactiae* | reduced virulence |
| gene2274 | Chr | tsr | PHI:6981 | *Salmonella enterica* | reduced virulence |
| gene2275 | Chr | bpdB | PHI:3646 | *Brucella melitensis* | reduced virulence |
| gene2279 | Chr | potA | PHI:6319 | *Streptococcus pneumoniae* | reduced virulence |
| gene2285 | Chr | CSH1 | PHI:419 | *Candida albicans* | reduced virulence |
| gene2314 | Chr | glcB | PHI:6309 | *Staphylococcus aureus* | reduced virulence |
| gene2319 | Chr | lplA1 | PHI:6512 | *Listeria monocytogenes* | reduced virulence |
| gene2320 | Chr | GzC2H048 | PHI:1385 | *Fusarium graminearum* | unaffected pathogenicity |
| gene2323 | Chr | potA | PHI:6319 | *Streptococcus pneumoniae* | reduced virulence |
| gene2330 | Chr | Vatr2 | PHI:3028 | *Clavibacter michiganensis* | reduced virulence |
| gene2332 | Chr | aer | PHI:6979 | *Salmonella enterica* | reduced virulence |
| gene2335 | Chr | potA | PHI:6319 | *Streptococcus pneumoniae* | reduced virulence |
| gene2336 | Chr | niuD | PHI:6849 | *Helicobacter pylori* | reduced virulence |
| gene2337 | Chr | niuD | PHI:6849 | *Helicobacter pylori* | reduced virulence |
| gene2339 | Chr | iutA | PHI:3297 | *Pantoea stewartii* | reduced virulence |
| gene2341 | Chr | aphB | PHI:5548 | *Vibrio cholerae* | reduced virulence |
| gene2346 | Chr | vep20 | PHI:3648 | *Vibrio vulnificus* | unaffected pathogenicity |
| gene2366 | Chr | MPD1 | PHI:413 | *Parastagonospora nodorum* | unaffected pathogenicity |
| gene2371 | Chr | potA | PHI:6319 | *Streptococcus pneumoniae* | reduced virulence |
| gene2376 | Chr | fleQ | PHI:3219 | *Xanthomonas oryzae* | unaffected pathogenicity |
| gene2381 | Chr | RovM | PHI:5089 | *Yersinia pestis* | reduced virulence |
| gene2389 | Chr | mcpB | PHI:6985 | *Salmonella enterica* | reduced virulence |
| gene2390 | Chr | LIP1 | PHI:541 | *Botrytis cinerea* | unaffected pathogenicity |
| gene2393 | Chr | ccpE | PHI:5242 | *Staphylococcus aureus* | increased virulence (hypervirulence) |
| gene2395 | Chr | FNR | PHI:4876 | *Escherichia coli* | reduced virulence |
| gene2405 | Chr | VprA | PHI:3482 | *Vibrio cholerae* | reduced virulence |
| gene2406 | Chr | TceSR | PHI:4834 | *Brucella melitensis* | reduced virulence |
| gene2410 | Chr | GzOB019 | PHI:1579 | *Fusarium graminearum* | unaffected pathogenicity |
| gene2417 | Chr | MgtC | PHI:4897 | *Pseudomonas aeruginosa* | reduced virulence |
| gene2419 | Chr | CshA | PHI:6510 | *Listeria monocytogenes* | unaffected pathogenicity |
| gene2424 | Chr | glcA | PHI:6308 | *Staphylococcus aureus* | reduced virulence |
| gene2431 | Chr | PKS1 | PHI:55 | *Cochliobolus heterostrophus* | reduced virulence |
| gene2437 | Chr | CcpA | PHI:4564 | *Enterococcus faecium* | reduced virulence |
| gene2438 | Chr | pafP | PHI:4631 | *Escherichia coli* | reduced virulence |
| gene2442 | Chr | zmpR | PHI:3008 | *Streptococcus pneumoniae* | unaffected pathogenicity |
| gene2444 | Chr | ybtQ | PHI:6913 | *Escherichia coli* | reduced virulence |
| gene2453 | Chr | MacB | PHI:3928 | *Salmonella enterica* | reduced virulence |
| gene2455 | Chr | expI | PHI:4179 | *Dickeya solani* | reduced virulence |
| gene2465 | Chr | AOX1 | PHI:199 | *Passalora fulva* | reduced virulence |
| gene2466 | Chr | MoSSADH | PHI:2145 | *Magnaporthe oryzae* | loss of pathogenicity |
| gene2476 | Chr | mliC | PHI:4981 | *Edwardsiella tarda* | reduced virulence |
| gene2479 | Chr | slyA | PHI:2678 | *Salmonella enterica* | reduced virulence |
| gene2481 | Chr | oqxA | PHI:6448 | *Klebsiella pneumoniae* | reduced virulence |
| gene2485 | Chr | aphB | PHI:5548 | *Vibrio cholerae* | reduced virulence |
| gene2487 | Chr | LptA | PHI:4910 | *Haemophilus ducreyi* | unaffected pathogenicity |
| gene2490 | Chr | GOX1 | PHI:414 | *Parastagonospora nodorum* | unaffected pathogenicity |
| gene2495 | Chr | SoxR | PHI:3092 | *Pantoea stewartii* | reduced virulence |
| gene2497 | Chr | CFAS | PHI:2643 | *Leishmania infantum* | reduced virulence |
| gene2502 | Chr | pykF | PHI:3134 | *Yersinia pseudotuberculosis* | reduced virulence |
| gene2508 | Chr | sufC | PHI:6894 | *Salmonella enterica* | unaffected pathogenicity |
| gene2509 | Chr | sufB | PHI:6893 | *Salmonella enterica* | unaffected pathogenicity |
| gene2512 | Chr | ybdB | PHI:6270 | *Escherichia coli* | lethal |
| gene2521 | Chr | niuD | PHI:6849 | *Helicobacter pylori* | reduced virulence |
| gene2522 | Chr | pstB | PHI:3412 | *Xanthomonas citri* | loss of pathogenicity |
| gene2526 | Chr | ABC4 | PHI:1017 | *Magnaporthe oryzae* | reduced virulence |
| gene2527 | Chr | gpx31 | PHI:4936 | *Candida albicans* | unaffected pathogenicity |
| gene2528 | Chr | niuD | PHI:6849 | *Helicobacter pylori* | reduced virulence |
| gene2530 | Chr | hupA | PHI:3074 | *Edwardsiella tarda* | reduced virulence |
| gene2544 | Chr | OxyR | PHI:3714 | *Escherichia coli* | reduced virulence |
| gene2549 | Chr | katE | PHI:6090 | *Acinetobacter nosocomialis* | increased virulence (hypervirulence) |
| gene2561 | Chr | argD | PHI:3126 | *Erwinia amylovora* | reduced virulence |
| gene2564 | Chr | ychO | PHI:6184 | *Escherichia coli* | reduced virulence |
| gene2570 | Chr | StpC | PHI:6324 | *Staphylococcus aureus* | unaffected pathogenicity |
| gene2582 | Chr | tssM | PHI:5090 | *Acinetobacter baumannii* | effector (plant avirulence determinant) |
| gene2594 | Chr | fruR | PHI:2672 | *Salmonella enterica* | reduced virulence |
| gene2602 | Chr | MntE | PHI:4652 | *Streptococcus pyogenes* | unaffected pathogenicity |
| gene2606 | Chr | FTT0673p/prsAp | PHI:4732 | *Francisella tularensis* | unaffected pathogenicity |
| gene2609 | Chr | DgcP | PHI:4663 | *Pseudomonas aeruginosa* | reduced virulence |
| gene2627 | Chr | tcp | PHI:6980 | *Salmonella enterica* | reduced virulence |
| gene2631 | Chr | pvdL | PHI:6991 | *Pseudomonas aeruginosa* | loss of pathogenicity |
| gene2634 | Chr | wcbT | PHI:5347 | *Burkholderia pseudomallei* | reduced virulence |
| gene2650 | Chr | CpxR | PHI:4523 | *Escherichia coli* | reduced virulence |
| gene2653 | Chr | frdA | PHI:2960 | *Edwardsiella ictaluri* | reduced virulence |
| gene2656 | Chr | tsr | PHI:6981 | *Salmonella enterica* | reduced virulence |
| gene2661 | Chr | treA | PHI:6630 | *Xanthomonas citri* | increased virulence (hypervirulence) |
| gene2676 | Chr | F-avi4330 | PHI:2895 | *Agrobacterium vitis* | loss of pathogenicity |
| gene2690 | Chr | pdhR | PHI:3135 | *Yersinia pseudotuberculosis* | reduced virulence |
| gene2710 | Chr | MoVPR | PHI:2143 | *Magnaporthe oryzae* | reduced virulence |
| gene2723 | Chr | phoP | PHI:2674 | *Salmonella enterica* | reduced virulence |
| gene2725 | Chr | PhoQ | PHI:4899 | *Salmonella enterica* | unaffected pathogenicity |
| gene2732 | Chr | MacB | PHI:3928 | *Salmonella enterica* | reduced virulence |
| gene2734 | Chr | DgcP | PHI:4663 | *Pseudomonas aeruginosa* | reduced virulence |
| gene2744 | Chr | sreR | PHI:6117 | *Xanthomonas oryzae* | unaffected pathogenicity |
| gene2758 | Chr | Flil | PHI:6995 | *Pseudomonas aeruginosa* | reduced virulence |
| gene2759 | Chr | filj | PHI:6463 | *Salmonella enterica* | reduced virulence |
| gene2762 | Chr | fliM | PHI:6194 | *Leptospira interrogans* | reduced virulence |
| gene2765 | Chr | yscR | PHI:627 | *Salmonella enterica* | reduced virulence |
| gene2766 | Chr | ssaS | PHI:628 | *Salmonella enterica* | reduced virulence |
| gene2768 | Chr | rcsA | PHI:4478 | *Erwinia amylovora* | loss of pathogenicity |
| gene2769 | Chr | trg | PHI:6982 | *Salmonella enterica* | reduced virulence |
| gene2774 | Chr | DipA | PHI:3042 | *Pseudomonas aeruginosa* | reduced virulence |
| gene2776 | Chr | Mfd | PHI:6483 | *Bacillus cereus* | loss of pathogenicity |
| gene2778 | Chr | Rv0392c | PHI:3629 | *Mycobacterium tuberculosis* | unaffected pathogenicity |
| gene2780 | Chr | NagZ | PHI:3282 | *Xanthomonas campestris* | reduced virulence |
| gene2788 | Chr | glcB | PHI:6309 | *Staphylococcus aureus* | reduced virulence |
| gene2794 | Chr | KSA1 | PHI:724 | *Fusarium graminearum* | unaffected pathogenicity |
| gene2796 | Chr | fabG1 | PHI:5271 | *Ralstonia solanacearum* | lethal |
| gene2797 | Chr | pamA | PHI:3090 | *Paenibacillus larvae* | reduced virulence |
| gene2804 | Chr | RNase E | PHI:3723 | *Salmonella enterica* | reduced virulence |
| gene2808 | Chr | flgK | PHI:6360 | *Pantoea ananatis* | reduced virulence |
| gene2834 | Chr | PA0423 | PHI:3166 | *Pseudomonas aeruginosa* | reduced virulence |
| gene2835 | Chr | spr0084 | PHI:3154 | *Streptococcus pneumoniae* | reduced virulence |
| gene2836 | Chr | PA3242 | PHI:3787 | *Pseudomonas aeruginosa* | increased virulence (hypervirulence) |
| gene2838 | Chr | yihW | PHI:6545 | *Salmonella enterica* | unaffected pathogenicity |
| gene2849 | Chr | HrpM | PHI:2699 | *Xanthomonas citri* | loss of pathogenicity |
| gene2850 | Chr | opgG | PHI:5564 | *Dickeya dadantii* | reduced virulence |
| gene2852 | Chr | PLD2 | PHI:5093 | *Acinetobacter baumannii* | reduced virulence |
| gene2857 | Chr | adeK | PHI:6388 | *Acinetobacter baumannii* | unaffected pathogenicity |
| gene2858 | Chr | oqxA | PHI:6448 | *Klebsiella pneumoniae* | reduced virulence |
| gene2867 | Chr | yihW | PHI:6545 | *Salmonella enterica* | unaffected pathogenicity |
| gene2874 | Chr | sirA | PHI:560 | *Salmonella enterica* | reduced virulence |
| gene2878 | Chr | RhlR | PHI:6748 | *Pseudomonas aeruginosa* | reduced virulence |
| gene2895 | Chr | Sdh1 | PHI:3914 | *Parastagonospora nodorum* | reduced virulence |
| gene2922 | Chr | OmpA2 | PHI:4160 | *Klebsiella pneumoniae* | reduced virulence |
| gene2924 | Chr | SrrAB | PHI:5466 | *Staphylococcus aureus* | reduced virulence |
| gene2930 | Chr | ABC4 | PHI:1017 | *Magnaporthe oryzae* | reduced virulence |
| gene2936 | Chr | ssuA | PHI:4028 | *Xanthomonas citri* | reduced virulence |
| gene2939 | Chr | potA | PHI:6319 | *Streptococcus pneumoniae* | reduced virulence |
| gene2942 | Chr | GzOB046 | PHI:1605 | *Fusarium graminearum* | unaffected pathogenicity |
| gene2943 | Chr | ompK36 | PHI:2872 | *Klebsiella pneumoniae* | reduced virulence |
| gene2944 | Chr | PsAAT3 | PHI:6261 | *Phytophthora sojae* | reduced virulence |
| gene2959 | Chr | ABC3 | PHI:1018 | *Magnaporthe oryzae* | loss of pathogenicity |
| gene2962 | Chr | himD | PHI:2673 | *Salmonella enterica* | reduced virulence |
| gene2963 | Chr | pnp | PHI:3878 | *Salmonella enterica* | reduced virulence |
| gene2965 | Chr | aroA | PHI:2624 | *Salmonella enterica* | reduced virulence |
| gene2974 | Chr | rarA | PHI:6447 | *Klebsiella pneumoniae* | reduced virulence |
| gene2977 | Chr | lrp | PHI:6497 | *Xenorhabdus nematophila* | reduced virulence |
| gene2978 | Chr | Trr1 | PHI:6470 | *Beauveria bassiana* | unaffected pathogenicity |
| gene2979 | Chr | ABC3 | PHI:1018 | *Magnaporthe oryzae* | loss of pathogenicity |
| gene2980 | Chr | ybtQ | PHI:6913 | *Escherichia coli* | reduced virulence |
| gene2985 | Chr | clpV?5 | PHI:5335 | *Burkholderia pseudomallei* | effector (plant avirulence determinant) |
| gene2986 | Chr | clpS/clpA | PHI:6212 | *Pseudomonas aeruginosa* | reduced virulence |
| gene2987 | Chr | CspV | PHI:6372 | *Vibrio cholerae* | unaffected pathogenicity |
| gene2996 | Chr | MacB | PHI:3928 | *Salmonella enterica* | reduced virulence |
| gene3004 | Chr | potC | PHI:6321 | *Streptococcus pneumoniae* | reduced virulence |
| gene3006 | Chr | potA | PHI:6319 | *Streptococcus pneumoniae* | reduced virulence |
| gene3029 | Chr | potA | PHI:6319 | *Streptococcus pneumoniae* | reduced virulence |
| gene3033 | Chr | dgcB | PHI:4633 | *Burkholderia glumae* | reduced virulence |
| gene3036 | Chr | adeK | PHI:6388 | *Acinetobacter baumannii* | unaffected pathogenicity |
| gene3042 | Chr | PtdA | PHI:4911 | *Haemophilus ducreyi* | unaffected pathogenicity |
| gene3043 | Chr | OmpX | PHI:6250 | *Escherichia coli* | reduced virulence |
| gene3050 | Chr | MacB | PHI:3928 | *Salmonella enterica* | reduced virulence |
| gene3084 | Chr | Avenacinase | PHI:24 | *Gaeumannomyces graminis* | unaffected pathogenicity |
| gene3094 | Chr | MacB | PHI:3928 | *Salmonella enterica* | reduced virulence |
| gene3095 | Chr | potA | PHI:6319 | *Streptococcus pneumoniae* | reduced virulence |
| gene3102 | Chr | Rv3232c | PHI:3634 | *Mycobacterium tuberculosis* | reduced virulence |
| gene3105 | Chr | CshA | PHI:6510 | *Listeria monocytogenes* | unaffected pathogenicity |
| gene3112 | Chr | tsr | PHI:6981 | *Salmonella enterica* | reduced virulence |
| gene3121 | Chr | potA | PHI:6319 | *Streptococcus pneumoniae* | reduced virulence |
| gene3124 | Chr | wcbT | PHI:5347 | *Burkholderia pseudomallei* | reduced virulence |
| gene3127 | Chr | argD | PHI:3126 | *Erwinia amylovora* | reduced virulence |
| gene3128 | Chr | RED1 | PHI:2839 | *Cochliobolus heterostrophus* | reduced virulence |
| gene3133 | Chr | potA | PHI:6319 | *Streptococcus pneumoniae* | reduced virulence |
| gene3135 | Chr | modA10 | PHI:5422 | *Haemophilus influenzae* | increased virulence (hypervirulence) |
| gene3138 | Chr | ABC3 | PHI:1018 | *Magnaporthe oryzae* | loss of pathogenicity |
| gene3140 | Chr | galK | PHI:6269 | *Escherichia coli* | reduced virulence |
| gene3145 | Chr | PhoP | PHI:6110 | *Salmonella enterica* | reduced virulence |
| gene3146 | Chr | rcsC11 | PHI:3011 | *Salmonella enterica* | unaffected pathogenicity |
| gene3161 | Chr | TolB | PHI:3137 | *Pseudomonas aeruginosa* | reduced virulence |
| gene3162 | Chr | TolA | PHI:3472 | *Escherichia coli* | reduced virulence |
| gene3175 | Chr | frdA | PHI:2960 | *Edwardsiella ictaluri* | reduced virulence |
| gene3190 | Chr | kdpE | PHI:6314 | *Pseudomonas syringae* | increased virulence (hypervirulence) |
| gene3196 | Chr | Fur | PHI:4887 | *Escherichia coli* | unaffected pathogenicity |
| gene3197 | Chr | HEX1 | PHI:4987 | *Candida albicans* | reduced virulence |
| gene3203 | Chr | aphB | PHI:5548 | *Vibrio cholerae* | reduced virulence |
| gene3204 | Chr | GUS1 | PHI:2521 | *Aspergillus fumigatus* | lethal |
| gene3205 | Chr | glcB | PHI:6309 | *Staphylococcus aureus* | reduced virulence |
| gene3206 | Chr | MoDeam | PHI:5472 | *Magnaporthe oryzae* | reduced virulence |
| gene3210 | Chr | AsnB | PHI:2964 | *Xanthomonas oryzae* | reduced virulence |
| gene3217 | Chr | ubiI | PHI:7028 | *Escherichia coli* | reduced virulence |
| gene3220 | Chr | YbeY | PHI:3059 | *Vibrio cholerae* | loss of pathogenicity |
| gene3222 | Chr | PA3984 | PHI:3782 | *Pseudomonas aeruginosa* | reduced virulence |
| gene3228 | Chr | ybtQ | PHI:6913 | *Escherichia coli* | reduced virulence |
| gene3238 | Chr | PA4485 | PHI:5448 | *Pseudomonas aeruginosa* | unaffected pathogenicity |
| gene3242 | Chr | lipA | PHI:6492 | *Acinetobacter baumannii* | reduced virulence |
| gene3243 | Chr | tatA | PHI:2415 | *Vibrio cholerae* | reduced virulence |
| gene3246 | Chr | CspV | PHI:6372 | *Vibrio cholerae* | unaffected pathogenicity |
| gene3250 | Chr | gntU | PHI:6700 | *Vibrio cholerae* | reduced virulence |
| gene3270 | Chr | potA | PHI:6319 | *Streptococcus pneumoniae* | reduced virulence |
| gene3275 | Chr | FMT | PHI:3999 | *Staphylococcus aureus* | reduced virulence |
| gene3276 | Chr | glcV | PHI:6142 | *Listeria monocytogenes* | reduced virulence |
| gene3284 | Chr | tsr | PHI:6981 | *Salmonella enterica* | reduced virulence |
| gene3285 | Chr | potA | PHI:6319 | *Streptococcus pneumoniae* | reduced virulence |
| gene3298 | Chr | fabG2 | PHI:5272 | *Ralstonia solanacearum* | reduced virulence |
| gene3300 | Chr | KSA1 | PHI:724 | *Fusarium graminearum* | unaffected pathogenicity |
| gene3316 | Chr | rpfR | PHI:5504 | *Cronobacter turicensis* | unaffected pathogenicity |
| gene3321 | Chr | fliC | PHI:6462 | *Salmonella enterica* | reduced virulence |
| gene3346 | Chr | ADE2 | PHI:196 | *Candida albicans* | reduced virulence |
| gene3347 | Chr | ADE2 | PHI:14 | *Cryptococcus neoformans* | reduced virulence |
| gene3349 | Chr | MacB | PHI:3928 | *Salmonella enterica* | reduced virulence |
| gene3361 | Chr | copA | PHI:5275 | *Vibrio tasmaniensis* | reduced virulence |
| gene3362 | Chr | copA | PHI:5275 | *Vibrio tasmaniensis* | reduced virulence |
| gene3366 | Chr | RovM | PHI:5089 | *Yersinia pestis* | reduced virulence |
| gene3372 | Chr | hemH | PHI:4734 | *Francisella tularensis* | unaffected pathogenicity |
| gene3378 | Chr | lhnR | PHI:3491 | *Agrobacterium vitis* | reduced virulence |
| gene3379 | Chr | htpG | PHI:6472 | *Escherichia coli* | reduced virulence |
| gene3383 | Chr | aphB | PHI:5548 | *Vibrio cholerae* | reduced virulence |
| gene3393 | Chr | adeI | PHI:6386 | *Acinetobacter baumannii* | unaffected pathogenicity |
| gene3394 | Chr | AcrB | PHI:2469 | *Erwinia amylovora* | reduced virulence |
| gene3404 | Chr | ymoA | PHI:3819 | *Yersinia pestis* | increased virulence (hypervirulence) |
| gene3411 | Chr | ybtP | PHI:6912 | *Escherichia coli* | reduced virulence |
| gene3412 | Chr | ybtP | PHI:6912 | *Escherichia coli* | reduced virulence |
| gene3425 | Chr | hupA | PHI:3074 | *Edwardsiella tarda* | reduced virulence |
| gene3426 | Chr | SrrAB | PHI:5466 | *Staphylococcus aureus* | reduced virulence |
| gene3427 | Chr | ClpX | PHI:3004 | *Staphylococcus aureus* | reduced virulence |
| gene3428 | Chr | ClpP | PHI:3040 | *Salmonella enterica* | reduced virulence |
| gene3429 | Chr | ClpX | PHI:3004 | *Staphylococcus aureus* | reduced virulence |
| gene3430 | Chr | ClpP | PHI:3040 | *Salmonella enterica* | reduced virulence |
| gene3436 | Chr | XAC1258 | PHI:4147 | *Xanthomonas citri* | reduced virulence |
| gene3442 | Chr | XC_3703 | PHI:3259 | *Xanthomonas campestris* | effector (plant avirulence determinant) |
| gene3449 | Chr | CSH1 | PHI:419 | *Candida albicans* | reduced virulence |
| gene3455 | Chr | nrdR | PHI:3464 | *Streptococcus pyogenes* | unaffected pathogenicity |
| gene3463 | Chr | TSA1 | PHI:386 | *Cryptococcus neoformans* | reduced virulence |
| gene3466 | Chr | BCMFS1 | PHI:544 | *Botrytis cinerea* | unaffected pathogenicity |
| gene3472 | Chr | stoS | PHI:6115 | *Xanthomonas oryzae* | unaffected pathogenicity |
| gene3473 | Chr | SrrAB | PHI:5466 | *Staphylococcus aureus* | reduced virulence |
| gene3496 | Chr | lacZ | PHI:6268 | *Escherichia coli* | reduced virulence |
| gene3498 | Chr | CspV | PHI:6372 | *Vibrio cholerae* | unaffected pathogenicity |
| gene3508 | Chr | ABC3 | PHI:1018 | *Magnaporthe oryzae* | loss of pathogenicity |
| gene3509 | Chr | potA | PHI:6319 | *Streptococcus pneumoniae* | reduced virulence |
| gene3537 | Chr | ompF | PHI:7023 | *Escherichia coli* | reduced virulence |
| gene3559 | Chr | MacB | PHI:3928 | *Salmonella enterica* | reduced virulence |
| gene3566 | Chr | iaaH-1 | PHI:4171 | *Pseudomonas savastanoi* | reduced virulence |
| gene3580 | Chr | ipx10 | PHI:2975 | *Pseudomonas syringae* | effector (plant avirulence determinant) |
| gene3591 | Chr | MacB | PHI:3928 | *Salmonella enterica* | reduced virulence |
| gene3612 | Chr | PA3644 | PHI:3785 | *Pseudomonas aeruginosa* | reduced virulence |
| gene3614 | Chr | PA3646 | PHI:3784 | *Pseudomonas aeruginosa* | reduced virulence |
| gene3631 | Chr | HtrA | PHI:6358 | *Haemophilus parasuis* | reduced virulence |
| gene3637 | Chr | CLC-A | PHI:286 | *Cryptococcus neoformans* | reduced virulence |
| gene3641 | Chr | potA | PHI:6319 | *Streptococcus pneumoniae* | reduced virulence |
| gene3642 | Chr | fiuA | PHI:6373 | *Pseudomonas aeruginosa* | reduced virulence |
| gene3647 | Chr | GzOB019 | PHI:1579 | *Fusarium graminearum* | unaffected pathogenicity |
| gene3649 | Chr | DksA | PHI:6508 | *Salmonella enterica* | reduced virulence |
| gene3654 | Chr | Pbl1 | PHI:2257 | *Parastagonospora nodorum* | unaffected pathogenicity |
| gene3655 | Chr | XC_2466 | PHI:3968 | *Xanthomonas campestris* | reduced virulence |
| gene3657 | Chr | ABC4 | PHI:1017 | *Magnaporthe oryzae* | reduced virulence |
| gene3658 | Chr | Can1 | PHI:673 | *Cryptococcus neoformans* | unaffected pathogenicity |
| gene3659 | Chr | XhpT | PHI:2936 | *Xylella fastidiosa* | reduced virulence |
| gene3660 | Chr | CBL1 | PHI:443 | *Fusarium graminearum* | reduced virulence |
| gene3661 | Chr | lac2 | PHI:2700 | *Colletotrichum orbiculare* | reduced virulence |
| gene3664 | Chr | SPE3-LYS9 | PHI:384 | *Cryptococcus neoformans* | loss of pathogenicity |
| gene3669 | Chr | CspV | PHI:6372 | *Vibrio cholerae* | unaffected pathogenicity |
| gene3670 | Chr | SidH | PHI:2322 | *Aspergillus fumigatus* | reduced virulence |
| gene3671 | Chr | Fox3p | PHI:3688 | *Candida albicans* | unaffected pathogenicity |
| gene3672 | Chr | cifA | PHI:2934 | *Pseudomonas cichorii* | reduced virulence |
| gene3677 | Chr | LssB | PHI:4975 | *Legionella pneumophila* | reduced virulence |
| gene3679 | Chr | LpdA | PHI:6520 | *Legionella pneumophila* | effector (plant avirulence determinant) |
| gene3682 | Chr | pdhR | PHI:3135 | *Yersinia pseudotuberculosis* | reduced virulence |
| gene3689 | Chr | ecf18 | PHI:2878 | *Pseudomonas syringae* | unaffected pathogenicity |
| gene3696 | Chr | pilA | PHI:6362 | *Pantoea ananatis* | reduced virulence |
| gene3697 | Chr | pilT | PHI:6363 | *Pantoea ananatis* | reduced virulence |
| gene3699 | Chr | IMPDH | PHI:4071 | *Streptococcus suis* | reduced virulence |
| gene3703 | Chr | PSPTO_2696 | PHI:3119 | *Pseudomonas syringae* | unaffected pathogenicity |
| gene3704 | Chr | VcpD | PHI:702 | *Vibrio cholerae* | reduced virulence |
| gene3705 | Chr | secA | PHI:5240 | *Acinetobacter baumannii* | reduced virulence |
| gene3708 | Chr | wcbS | PHI:5346 | *Burkholderia pseudomallei* | reduced virulence |
| gene3722 | Chr | rsmH | PHI:4961 | *Staphylococcus aureus* | reduced virulence |
| gene3724 | Chr | fruR | PHI:2672 | *Salmonella enterica* | reduced virulence |
| gene3725 | Chr | MoIlv6 | PHI:3976 | *Magnaporthe oryzae* | loss of pathogenicity |
| gene3726 | Chr | MoIlv2 | PHI:3975 | *Magnaporthe oryzae* | loss of pathogenicity |
| gene3729 | Chr | LEU2 | PHI:504 | *Saccharomyces cerevisiae* | reduced virulence |
| gene3737 | Chr | potA | PHI:6319 | *Streptococcus pneumoniae* | reduced virulence |
| gene3746 | Chr | RsmA | PHI:2735 | *Pectobacterium wasabiae* | increased virulence (hypervirulence) |
| gene3762 | Chr | nhaR | PHI:3267 | *Escherichia coli* | reduced virulence |
| gene3763 | Chr | nhaA | PHI:3266 | *Escherichia coli* | reduced virulence |
| gene3764 | Chr | DnaJ | PHI:6986 | *Streptococcus pneumoniae* | reduced virulence |
| gene3780 | Chr | FVEG_12521 | PHI:3389 | *Fusarium verticillioides* | unaffected pathogenicity |
| gene3783 | Chr | arcA | PHI:6532 | *Escherichia coli* | reduced virulence |
| gene3791 | Chr | GzC2H048 | PHI:1385 | *Fusarium graminearum* | unaffected pathogenicity |
| gene3796 | Chr | ABC4 | PHI:1017 | *Magnaporthe oryzae* | reduced virulence |
| gene3797 | Chr | pstB | PHI:3412 | *Xanthomonas citri* | loss of pathogenicity |
| gene3798 | Chr | cipA | PHI:4837 | *Pseudomonas cichorii* | reduced virulence |
| gene3801 | Chr | ABC4 | PHI:1017 | *Magnaporthe oryzae* | reduced virulence |
| gene3802 | Chr | aphB | PHI:5548 | *Vibrio cholerae* | reduced virulence |
| gene3839 | Chr | NPS1 | PHI:3659 | *Fusarium graminearum* | unaffected pathogenicity |
| gene3845 | Chr | CcpA | PHI:4564 | *Enterococcus faecium* | reduced virulence |
| gene3849 | Chr | PKS4 (ZEA1) | PHI:714 | *Fusarium graminearum* | unaffected pathogenicity |
| gene3880 | Chr | Can2 | PHI:674 | *Cryptococcus neoformans* | unaffected pathogenicity |
| gene3882 | Chr | gntU | PHI:6700 | *Vibrio cholerae* | reduced virulence |
| gene3883 | Chr | fabG1 | PHI:5271 | *Ralstonia solanacearum* | lethal |
| gene3923 | Chr | rsmI | PHI:4960 | *Staphylococcus aureus* | reduced virulence |
| gene3943 | Chr | Clr3 | PHI:5036 | *Ustilago maydis* | reduced virulence |
| gene3944 | Chr | cheM | PHI:6983 | *Salmonella enterica* | reduced virulence |
| gene3954 | Chr | cpdB | PHI:7020 | *Escherichia coli* | reduced virulence |
| gene3964 | Chr | tsr | PHI:6981 | *Salmonella enterica* | reduced virulence |
| gene3968 | Chr | ClaSSD1 | PHI:862 | *Colletotrichum lagenarium* | loss of pathogenicity |
| gene3970 | Chr | purA | PHI:2625 | *Salmonella enterica* | reduced virulence |
| gene3975 | Chr | hfq | PHI:6403 | *Yersinia enterocolitica* | reduced virulence |
| gene3976 | Chr | CKS1 | PHI:6158 | *Magnaporthe oryzae* | reduced virulence |
| gene3977 | Chr | GzssDB001 | PHI:1616 | *Fusarium graminearum* | unaffected pathogenicity |
| gene3984 | Chr | bpaC | PHI:4668 | *Burkholderia pseudomallei* | reduced virulence |
| gene3987 | Chr | RovM | PHI:5089 | *Yersinia pestis* | reduced virulence |
| gene3990 | Chr | Orn | PHI:6274 | *Pseudomonas aeruginosa* | reduced virulence |
| gene3995 | Chr | frdA | PHI:2960 | *Edwardsiella ictaluri* | reduced virulence |
| gene4001 | Chr | EF-P | PHI:6420 | *Salmonella enterica* | increased virulence (hypervirulence) |
| gene4009 | Chr | GroEL | PHI:3085 | *Porphyromonas gingivalis* | increased virulence (hypervirulence) |
| gene4033 | Chr | T6SS1 | PHI:4558 | *Escherichia coli* | effector (plant avirulence determinant) |
| gene4038 | Chr | T6SS2 | PHI:4559 | *Escherichia coli* | effector (plant avirulence determinant) |
| gene4049 | Chr | fiuA | PHI:6373 | *Pseudomonas aeruginosa* | reduced virulence |
| gene4050 | Chr | aphB | PHI:5548 | *Vibrio cholerae* | reduced virulence |
| gene4064 | Chr | treA | PHI:6630 | *Xanthomonas citri* | increased virulence (hypervirulence) |
| gene4072 | Chr | aphB | PHI:5548 | *Vibrio cholerae* | reduced virulence |
| gene4075 | Chr | ABC4 | PHI:1017 | *Magnaporthe oryzae* | reduced virulence |
| gene4089 | Chr | DipA | PHI:3042 | *Pseudomonas aeruginosa* | reduced virulence |
| gene4099 | Chr | aer | PHI:6979 | *Salmonella enterica* | reduced virulence |
| gene4107 | Chr | ccpE | PHI:5242 | *Staphylococcus aureus* | increased virulence (hypervirulence) |
| gene4113 | Chr | ramA | PHI:5370 | *Salmonella enterica* | reduced virulence |
| gene4114 | Chr | bpdA | PHI:3645 | *Brucella melitensis* | loss of pathogenicity |
| gene4139 | Chr | XacFhaB | PHI:5388 | *Xanthomonas citri* | reduced virulence |
| gene4141 | Chr | SSBX | PHI:2737 | *Xanthomonas oryzae* | reduced virulence |
| gene4148 | Chr | fruR | PHI:2672 | *Salmonella enterica* | reduced virulence |
| gene4149 | Chr | potA | PHI:6319 | *Streptococcus pneumoniae* | reduced virulence |
| gene4152 | Chr | orf245 | PHI:611 | *Salmonella enterica* | reduced virulence |
| gene4179 | Chr | FVEG_12521 | PHI:3389 | *Fusarium verticillioides* | unaffected pathogenicity |
| gene4184 | Chr | kdgR | PHI:4175 | *Dickeya solani* | unaffected pathogenicity |
| gene4186 | Chr | ICL1 | PHI:477 | *Candida albicans* | reduced virulence |
| gene4187 | Chr | Mls1 | PHI:365 | *Parastagonospora nodorum* | loss of pathogenicity |
| gene4195 | Chr | ADE5 | PHI:744 | *Fusarium graminearum* | reduced virulence |
| gene4197 | Chr | hupA | PHI:3074 | *Edwardsiella tarda* | reduced virulence |
| gene4208 | Chr | yedX | PHI:2651 | *Salmonella enterica* | unaffected pathogenicity |
| gene4238 | Chr | Pot13p | PHI:3689 | *Candida albicans* | unaffected pathogenicity |
| gene4243 | Chr | tatC | PHI:2417 | *Vibrio cholerae* | reduced virulence |
| gene4244 | Chr | tatB | PHI:2416 | *Vibrio cholerae* | reduced virulence |
| gene4245 | Chr | tatA | PHI:2415 | *Vibrio cholerae* | reduced virulence |
| gene4252 | Chr | MSY1 | PHI:442 | *Fusarium graminearum* | reduced virulence |
| gene4264 | Chr | corA | PHI:2479 | *Pectobacterium carotovorum* | reduced virulence |
| gene4271 | Chr | cyaA | PHI:2950 | *Pseudomonas syringae* | reduced virulence |
| gene4289 | Chr | algD | PHI:7031 | *Pseudomonas syringae* | unaffected pathogenicity |
| gene4293 | Chr | esaN | PHI:4613 | *Edwardsiella tarda* | increased virulence (hypervirulence) |
| gene4294 | Chr | thioredoxin 1 | PHI:2644 | *Salmonella enterica* | reduced virulence |
| gene4295 | Chr | CshA | PHI:6510 | *Listeria monocytogenes* | unaffected pathogenicity |
| gene4298 | Chr | fabG1 | PHI:5271 | *Ralstonia solanacearum* | lethal |
| gene4300 | Chr | PrsA2 | PHI:6137 | *Listeria monocytogenes* | reduced virulence |
| gene4302 | Chr | SoxR | PHI:3092 | *Pantoea stewartii* | reduced virulence |
| gene4303 | Chr | MoIlv1 | PHI:3257 | *Magnaporthe oryzae* | reduced virulence |
| gene4304 | Chr | edd | PHI:6698 | *Vibrio cholerae* | reduced virulence |
| gene4307 | Chr | MoIlv2 | PHI:3975 | *Magnaporthe oryzae* | loss of pathogenicity |
| gene4311 | Chr | RovM | PHI:5089 | *Yersinia pestis* | reduced virulence |
| gene4319 | Chr | ireA | PHI:6654 | *Escherichia coli* | unaffected pathogenicity |
| gene4324 | Chr | SoxR | PHI:3092 | *Pantoea stewartii* | reduced virulence |
| gene4326 | Chr | argH | PHI:2632 | *Staphylococcus aureus* | reduced virulence |
| gene4327 | Chr | MoARG1 | PHI:5232 | *Magnaporthe oryzae* | loss of pathogenicity |
| gene4333 | Chr | CBL1 | PHI:443 | *Fusarium graminearum* | reduced virulence |
| gene4334 | Chr | metJ | PHI:2695 | *Pectobacterium atrosepticum* | reduced virulence |
| gene4338 | Chr | fruR | PHI:2672 | *Salmonella enterica* | reduced virulence |
| gene4355 | Chr | Mqo | PHI:6190 | *Staphylococcus aureus* | reduced virulence |
| gene4360 | Chr | fabG1 | PHI:5271 | *Ralstonia solanacearum* | lethal |
| gene4361 | Chr | aphB | PHI:5548 | *Vibrio cholerae* | reduced virulence |
| gene4368 | Chr | CpxR | PHI:4523 | *Escherichia coli* | reduced virulence |
| gene4369 | Chr | envZ | PHI:2686 | *Salmonella enterica* | reduced virulence |
| gene4376 | Chr | envC | PHI:5573 | *Salmonella enterica* | reduced virulence |
| gene4379 | Chr | wcbT | PHI:5347 | *Burkholderia pseudomallei* | reduced virulence |
| gene4386 | Chr | Lmepi | PHI:1133 | *Leptosphaeria maculans* | loss of pathogenicity |
| gene4390 | Chr | cps2G | PHI:6139 | *Streptococcus suis* | reduced virulence |
| gene4393 | Chr | cps2J | PHI:6140 | *Streptococcus suis* | reduced virulence |
| gene4401 | Chr | radC | PHI:4701 | *Streptococcus pneumoniae* | reduced virulence |
| gene4405 | Chr | URA5 | PHI:687 | *Cryptococcus neoformans* | reduced virulence |
| gene4410 | Chr | ccpE | PHI:5242 | *Staphylococcus aureus* | increased virulence (hypervirulence) |
| gene4418 | Chr | MoAcat1 | PHI:5082 | *Magnaporthe oryzae* | reduced virulence |
| gene4422 | Chr | rcsB | PHI:2499 | *Erwinia amylovora* | loss of pathogenicity |
| gene4427 | Chr | relA | PHI:6720 | *Streptococcus suis* | reduced virulence |
| gene4429 | Chr | Mfd | PHI:6483 | *Bacillus cereus* | loss of pathogenicity |
| gene4440 | Chr | barA | PHI:562 | *Salmonella enterica* | unaffected pathogenicity |
| gene4441 | Chr | LuxO | PHI:708 | *Vibrio cholerae* | reduced virulence |
| gene4458 | Chr | CcpA | PHI:4564 | *Enterococcus faecium* | reduced virulence |
| gene4459 | Chr | orf408 | PHI:612 | *Salmonella enterica* | reduced virulence |
| gene4462 | Chr | StpC | PHI:6324 | *Staphylococcus aureus* | unaffected pathogenicity |
| gene4464 | Chr | fabG1 | PHI:5271 | *Ralstonia solanacearum* | lethal |
| gene4469 | Chr | QseB | PHI:6882 | *Salmonella enterica* | increased virulence (hypervirulence) |
| gene4470 | Chr | qseC | PHI:3709 | *Pectobacterium carotovorum* | reduced virulence |
| gene4492 | Chr | GFA1 | PHI:2513 | *Aspergillus fumigatus* | lethal |
| gene4495 | Chr | GNO1 | PHI:668 | *Cryptococcus neoformans* | unaffected pathogenicity |
| gene4498 | Chr | Rv0930 | PHI:3635 | *Mycobacterium tuberculosis* | unaffected pathogenicity |
| gene4499 | Chr | pstB | PHI:3412 | *Xanthomonas citri* | loss of pathogenicity |
| p_gene0006 | p | hns | PHI:6369 | *Escherichia coli* | increased virulence (hypervirulence) |
| p_gene0035 | p | acrB | PHI:6451 | *Klebsiella pneumoniae* | reduced virulence |
| p_gene0036 | p | AcrD | PHI:4043 | *Erwinia amylovora* | unaffected pathogenicity |
| p_gene0049 | p | xrp8 | PHI:4137 | *Xanthomonas oryzae* | effector (plant avirulence determinant) |
| p_gene0053 | p | xrp8 | PHI:4137 | *Xanthomonas oryzae* | effector (plant avirulence determinant) |
| p_gene0057 | p | MorA | PHI:4684 | *Pseudomonas aeruginosa* | increased virulence (hypervirulence) |
| p_gene0058 | p | fabG1 | PHI:5271 | *Ralstonia solanacearum* | lethal |
| p_gene0064 | p | Abd1 | PHI:2255 | *Parastagonospora nodorum* | unaffected pathogenicity |
| p_gene0067 | p | BdlA | PHI:3041 | *Pseudomonas aeruginosa* | reduced virulence |
| p_gene0068 | p | sagB | PHI:3436 | *Streptococcus pyogenes* | unaffected pathogenicity |
| p_gene0079 | p | MorA | PHI:4684 | *Pseudomonas aeruginosa* | increased virulence (hypervirulence) |
| p_gene0093 | p | MoSSADH | PHI:2145 | *Magnaporthe oryzae* | loss of pathogenicity |
| p_gene0108 | p | potA | PHI:6319 | *Streptococcus pneumoniae* | reduced virulence |
| p_gene0110 | p | MacB | PHI:3928 | *Salmonella enterica* | reduced virulence |
| p_gene0115 | p | msrA2 | PHI:4580 | *Staphylococcus aureus* | unaffected pathogenicity |
| p_gene0139 | p | potA | PHI:6319 | *Streptococcus pneumoniae* | reduced virulence |
| p_gene0147 | p | hns | PHI:6369 | *Escherichia coli* | increased virulence (hypervirulence) |
|  |  |  |  |  |  |
