# Supplementary material for: Seed-Borne Erwinia persicina Affects the Growth and Physiology of Alfalfa (Medicago sativa L.)
Source: Front Microbiol. 2022 May 26;13:891188. doi: 10.3389/fmicb.2022.891188 (PMC9178255; doi:10.3389/fmicb.2022.891188)
Supplement: Supplementary file 3 [file Table_3.DOCX]

**TABLE S3 |** Results of the ANOVA conducted for the treatment type and host tissue

| **Physio-biochemical**  **parameters** | **Treatment types (TT)** | | | **Host tissues (HT)** | | | **TT×HT** | | |
| --- | --- | --- | --- | --- | --- | --- | --- | --- | --- |
|  | ***F-*value** | ***P-*value** | ***η^2^*** | ***F-*value** | ***P-*value** | ***η^2^*** | ***F-*value** | ***P-*value** | ***η^2^*** |
| SOD | 10.091 | 0.008 | 0.457 | 675.412 | < 0.001 | 0.991 | 555.131 | < 0.001 | 0.989 |
| POD | 689.662 | < 0.001 | 0.983 | 485.802 | < 0.001 | 0.988 | 69.937 | < 0.001 | 0.921 |
| APX | 638.129 | < 0.001 | 0.982 | 879.066 | < 0.001 | 0.993 | 108.830 | < 0.001 | 0.948 |
| CAT | 0.055 | 0.818 | 0.005 | 363.029 | < 0.001 | 0.984 | 198.055 | < 0.001 | 0.971 |
| SP | 345.268 | < 0.001 | 0.966 | 21864.264 | < 0.001 | 1.000 | 901.305 | < 0.001 | 0.993 |
| SS | 2.872 | 0.116 | 0.193 | 1334.129 | < 0.001 | 0.996 | 63.545 | < 0.001 | 0.914 |
| PAL | 39.519 | < 0.001 | 0.767 | 1575.544 | < 0.001 | 0.996 | 80.988 | < 0.001 | 0.931 |
| PPO | 308.133 | < 0.001 | 0.963 | 456.046 | < 0.001 | 0.987 | 15.323 | < 0.001 | 0.719 |
| MDA | 0.722 | 0.412 | 0.057 | 1525.623 | < 0.001 | 0.996 | 150.674 | < 0.001 | 0.962 |

*η^2^ represents the effect size.*
