# Supplementary material for: Seed-Borne Erwinia persicina Affects the Growth and Physiology of Alfalfa (Medicago sativa L.)
Source: Front Microbiol. 2022 May 26;13:891188. doi: 10.3389/fmicb.2022.891188 (PMC9178255; doi:10.3389/fmicb.2022.891188)
Supplement: Supplementary file 4 [file Table_4.DOCX]

**TABLE S4 |** Physio-biochemical indicators (PI) loadings and contributions for PC1

| **Tissue** | **PI** | **Loadings** | **Contributions** | **Rank** |
| --- | --- | --- | --- | --- |
| Root | SOD | 0.9964 | 11.3089 | 1^†††^ |
|  | POD | 0.9961 | 11.3032 | 2^†††^ |
|  | SP | 0.9943 | 11.2605 | 3^†††^ |
|  | MDA | 0.9917 | 11.2019 | 4^††^ |
|  | CAT | -0.9909 | 11.1835 | 5^††^ |
|  | PPO | 0.9890 | 11.1425 | 6^††^ |
|  | SS | -0.9875 | 11.1080 | 7^††^ |
|  | APX | 0.9862 | 11.0790 | 8^†^ |
|  | PAL | 0.9561 | 10.4126 | 9^†^ |
| Stem | SP | -0.9977 | 12.8958 | 1^†††^ |
|  | APX | 0.9893 | 12.6777 | 2^†††^ |
|  | POD | 0.9865 | 12.6060 | 3^†††^ |
|  | PPO | 0.9539 | 11.7890 | 4^††^ |
|  | CAT | -0.9209 | 10.9873 | 5^††^ |
|  | SOD | 0.9142 | 10.8277 | 6^††^ |
|  | SS | 0.9093 | 10.7110 | 7^††^ |
|  | MDA | -0.8415 | 9.1725 | 8^†^ |
|  | PAL | -0.8020 | 8.3329 | 9^†^ |
| Leaf | SP | -0.9973 | 12.0445 | 1^†††^ |
|  | SOD | -0.9899 | 11.8653 | 2^†††^ |
|  | MDA | -0.9831 | 11.7049 | 3^†††^ |
|  | CAT | 0.9824 | 11.6878 | 4^††^ |
|  | PAL | -0.9819 | 11.6744 | 5^††^ |
|  | PPO | 0.9627 | 11.2236 | 6^††^ |
|  | POD | 0.9453 | 10.8213 | 7^††^ |
|  | APX | 0.9316 | 10.5106 | 8^†^ |
|  | SS | 0.8362 | 8.4676 | 9^†^ |

*†††: extremely important (75% ≤ contribution ≤ 100%), ††: important (25% ≤ contribution < 75%), †: unimportant (0% ≤ contri-bution < 25%)*
